# Artificial Intelligence Is Stereotypically Linked More with Socially Dominant Groups in Natural Language

**DOI:** 10.1002/advs.202508623

**Published:** 2025-07-28

**Authors:** Zixi Wang, Haodong Xia, Han Wu Shuang Bao, Yiming Jing, Ruolei Gu

**Affiliations:** ^1^ State Key Laboratory of Cognitive Science and Mental Health Institute of Psychology Chinese Academy of Sciences Beijing 100101 China; ^2^ Department of Psychology University of Chinese Academy of Sciences Beijing 100049 China; ^3^ School of Psychology and Cognitive Science East China Normal University Shanghai 200062 China

**Keywords:** artificial intelligence, natural language processing, social representation, stereotype

## Abstract

Despite the increasingly important role that artificial intelligence (AI) plays in society, its social representation remains underexplored. This study reveals that AI is asymmetrically stereotyped, being more closely associated with socially dominant groups. This conclusion is based on these investigations of AI's associations along the warmth–competence dimensions of the stereotype content model, its connections with socially advantaged (e.g., men, young, rich, white) versus disadvantaged (e.g., women, old, poor, non‐white) demographic groups, and its perceived impact on high‐ versus low‐prestige occupations. Across four studies using language‐based analyses with static word embeddings, BERT‐based models, and GPT‐4o, as well as human‐participant experiment validation, it is found that i) AI is strongly associated with high competence but exhibits variability in the warmth dimension (Study 1); ii) AI is more closely linked to advantaged demographic groups (Study 2); iii) advantaged demographic groups are semantically closer to AI than their disadvantaged counterparts along the warmth–competence dimensions (Study 3); and iv) high‐prestige occupations (rather than low‐prestige ones) are associated more strongly with AI's benefits than with its threats. Together, these findings indicate that public perceptions of AI are systematically biased toward socially dominant groups, potentially reinforcing existing social inequalities and raising concerns about an emerging “AI divide.”

## Graphical abstract

AI is not seen through an unbiased lens. People tend to stereotype AI as competent and link it with socially advantaged groups—such as men, the wealthy, the young, and prestigious occupations—raising concerns that such perceptions may deepen existing social divides rather than bridge them.

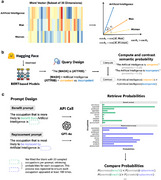

## 1 Introduction

Artificial intelligence (AI) techniques, particularly generative AI, are rapidly integrating into daily life as AI agents assume increasingly complex and diverse social roles.^[^ ^1^, ^2^ ^]^ In evaluating the societal impact of these technologies, it is essential to recognize that social representations of AI are not unbiased. Recent studies suggest that people's perceptions of AI are systematically influenced by pre‐existing stereotypes and social frameworks, which substantially affect the societal understanding and adoption of AI technologies.^[^ ^3^, ^4^ ^]^

First, individuals tend to position AI along social stereotype dimensions, even when interacting with AI systems specifically designed to maintain neutrality (e.g., generative AI programmed to respond with objective, impartial outputs).^[^ ^5^, ^6^, ^7^ ^]^ This tendency is possibly driven by the anthropomorphism effect, wherein people attribute human‐like characteristics to non‐human entities.^[^ ^8^, ^9^ ^]^ According to the stereotype content model (SCM),^[^ ^10^, ^11^, ^12^ ^]^ perceptions of individuals and groups are primarily evaluated along two dimensions: *warmth* (e.g., “Is this target a friend or a foe?”) and *competence* (e.g., “Can this target act on their intentions?”). Notably, this framework extends beyond human social cognition and influences psychological responses to representations and evaluations of AI systems.^[^ ^13^ ^]^ Recent large‐scale surveys reveal that the dimensions of warmth and competence not only shape public perceptions of AI but also predict technology adoption intentions, although findings remain inconsistent regarding whether AI agents are perceived as high or low on these dimensions.^[^ ^14^, ^15^ ^]^ Second, AI is often disproportionately associated with demographic advantaged groups in people's minds. That is, male, younger, higher‐educated, and urban individuals, compared to their demographic counterparts, are consistently perceived as more capable of engaging with AI technologies, such as autonomous systems and robotics.^[^ ^16^, ^17^, ^18^, ^19^ ^]^

The above stereotype‐based disparities in AI representations have the potential to exacerbate unequal adoption rates of AI technologies. Some populations may perceive that AI “is not their thing,” deeming the technology incompatible with their group identity or social standing.^[^ ^20^ ^]^ An “AI divide” could then emerge, whereby certain groups are less likely to benefit from educational, employment, investment, and medical opportunities arising from AI advancements, thus amplifying existing socioeconomic inequalities.^[^ ^21^, ^22^, ^23^ ^]^ Empirical evidence supports these concerns, revealing that early adopters of cutting‐edge AI technologies are predominantly young, male, and urban‐based.^[^ ^24^ ^]^ For example, significant gender gaps persist across various communities, with females more likely than males to refrain from using generative AI.^[^ ^24^, ^25^, ^26^ ^]^ Similarly, age‐based differences are evident in the entrepreneurial sector, where younger startup founders in the United States exhibit greater interest in integrating generative AI into their businesses compared to older entrepreneurs.^[^ ^23^ ^]^

Given the profound societal implications, it is crucial to systematically investigate the stereotypical representations of AI for more equitable access to its benefits.^[^ ^20^, ^27^, ^28^ ^]^ Compared to previous work, the major contributions of this study are twofold: Theoretically, while prior research has mapped AI onto abstract dimensions of warmth and competence, our research offers a more concrete and socially‐grounded characterization. Guided by the SCM, which posits that perceived competence stems from social status,^[^ ^29^, ^30^ ^]^ we test whether AI's perceived competence is aligned with its psychological associations with high‐status social groups. Methodologically, recent research on this topic has predominantly relied on surveys and questionnaires,^[^ ^14^, ^15^, ^19^, ^24^, ^26^ ^]^ which are subject to limitations such as social desirability bias^[^ ^18^ ^]^ and systematic sampling biases. To overcome these limitations, we turned to natural language — a rich repository of societal beliefs embedded in billions of linguistic expressions. Advances in natural language processing (NLP) now enable researchers to uncover shared social representations from large‐scale corpora with high ecological validity and generalizability.^[^ ^31^, ^32^, ^33^, ^34^ ^]^

**Figure 1:**
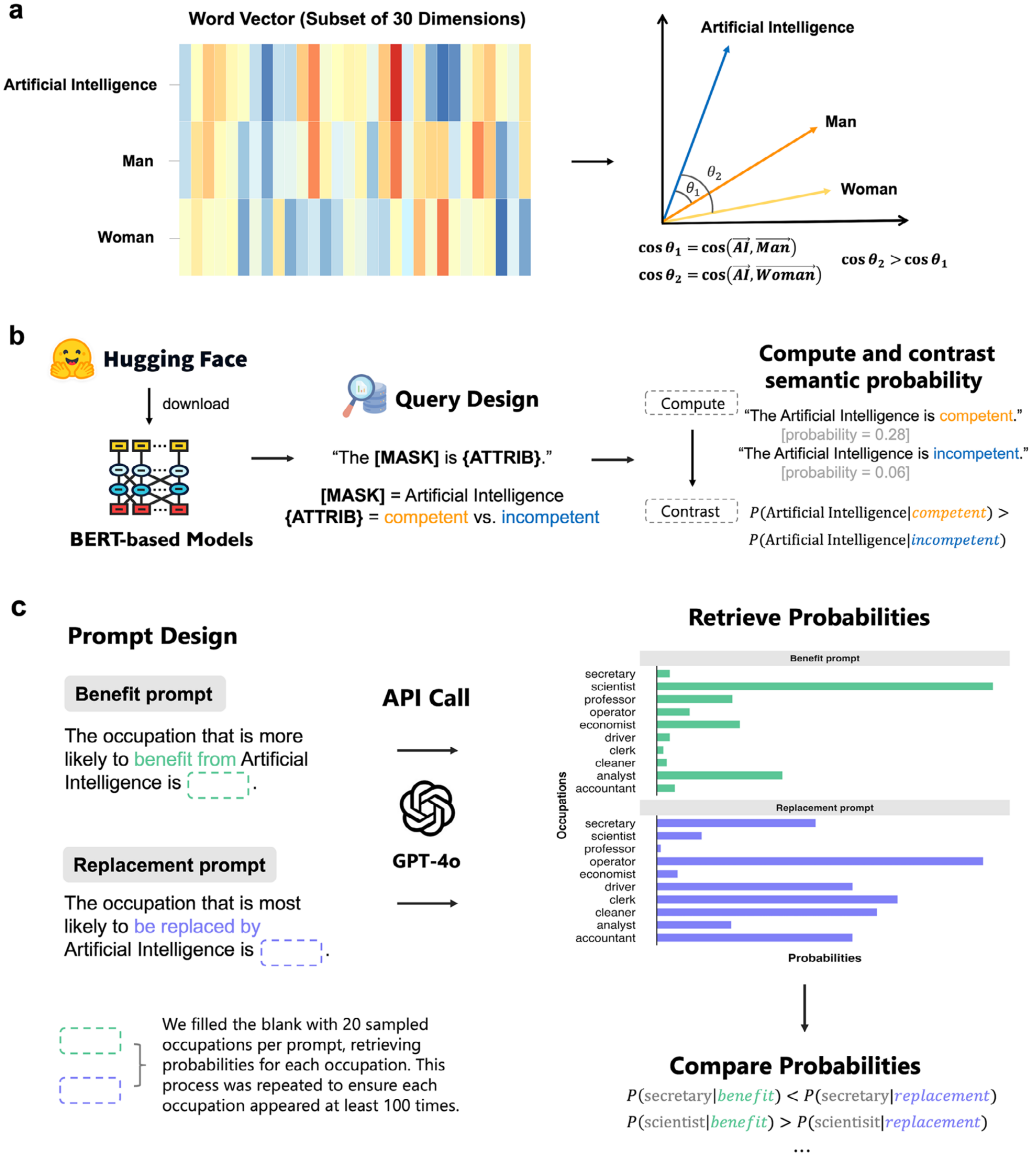
Workflow of the text‐analytic methods used in the paper. *Note*. a) Single‐Category Word Embedding Association Test (SC‐WEAT) using word embeddings. b) Fill‐Mask Association Test (FMAT) employing BERT‐based models. c) Probing analysis utilizing the GPT‐4o model.

To this end, we employed a tiered approach to text analysis by integrating three complementary NLP techniques (see **Figure** **1**): word embedding models, BERT language models, and the GPT‐4o model. First, we used static word embeddings to analyze word‐level semantic relationships within medium‐scale corpora. Words are represented as high‐dimensional vectors,^[^ ^35^ ^]^ and their relative semantic proximity can be quantified using the Single‐Category Word Embedding Association Test (SC‐WEAT),^[^ ^36^ ^]^ which computes the average cosine similarity between target words and contrasting attribute sets.^[^ ^37^ ^]^ This approach has been empirically validated for detecting shared social representations embedded in language.^[^ ^33^, ^37^, ^38^ ^]^ Next, we employed BERT language models pretrained using Masked Language Modeling (MLM), which can predict masked words based on surrounding context, similar to a cloze test.^[^ ^39^ ^]^ This enables sentence‐level analysis of context‐sensitive meanings. Building on this architecture, the Fill‐Mask Association Test (FMAT)^[^ ^40^ ^]^ estimates the relative likelihood of target words appearing in masked sentence positions, which has been validated for measuring social stereotypes.^[^ ^40^, ^41^ ^]^ Finally, we leveraged GPT‐4o, a large‐scale generative language model that captures discourse‐level nuances more effectively than static word embeddings or BERT models, owing to its vast training data and architectural complexity. This allows the model to better represent complex semantic relationships in the real world and to more closely approximate natural human language use, exceeding the ecological validity of prior NLP approaches.^[^ ^39^ ^]^ To examine the stereotypical representation of AI in GPT‐4o's outputs, we adapted a probing method proposed by Hofmann et al.,^[^ ^42^ ^]^ originally developed to assess racial biases. Together, these three methods form a progressive framework that improves the depth, contextuality, and ecological validity of our semantic insights.

Additionally, to further validate our findings from natural language analysis, we incorporated behavioral measures using the Single‐Category Implicit Association Test (SC‐IAT) and its corresponding explicit assessments.^[^ ^43^ ^]^ The SC‐IAT, a widely used paradigm for investigating implicit stereotypes and biases, is a modified version of the traditional IAT procedure.^[^ ^44^ ^]^

Using these approaches, we investigated a central question: Is AI stereotypically associated with socially dominant groups—those who hold greater status, influence, and access to resources?^[^ ^45^ ^]^ Specifically, we examined i) whether AI is perceived as high or low in competence and warmth, ii) whether it is more closely associated with advantaged demographic groups (e.g., men, younger people, wealthier individuals, and white people), and iii) whether it is perceived to benefit high‐prestige occupations more than low‐prestige ones. Based on these considerations, we proposed the following hypotheses (see **Table** **1** for an overview of studies):

**Table 1:** Overview of the studies in the paper.

Study	Method	Results (AI associations)
*Study 1: Association between AI and High versus Low Competence and Warmth*		
1a	SC‐WEAT (word embeddings)	high > low competence; high > low warmth
1b	FMAT (BERT models)	high > low competence; low > high warmth
Supplementary Study 1	SC‐IAT and self‐report measures (human participants)	high > low competence; high > low warmth
*Study 2: Association between AI and Advantaged versus Disadvantaged Demographic Groups*		
2a	SC‐WEAT (word embeddings)	Advantaged > disadvantaged demographic groups
2b	FMAT (BERT models)	Advantaged > disadvantaged demographic groups
Supplementary Study 2	SC‐IAT and self‐report measures (human participants)	Advantaged > disadvantaged demographic groups
*Study 3: Distances between AI and Demographic Groups in the Competence–Warmth Space*		
Study 3	SC‐WEAT (word embeddings) and FMAT (BERT models)	Along the competence–warmth dimension, AI's distribution patterns align closely with advantaged > disadvantaged demographic groups.
*Study 4: Perceived Benefit vs. Threat of AI across Occupations of Varying Prestige*		
Study 4	Probing analysis (GPT‐4o)	AI's positive impact is associated with high > low prestigious occupations.

**Hypothesis 1**. Social perceptions of AI would reflect warmth–competence stereotypes, though their direction may vary based on prior mixed findings.

**Hypothesis 2**. AI would be more strongly associated with advantaged demographic groups than with disadvantaged ones.

**Hypothesis 3**. High‐prestige occupations would be more likely to be perceived as benefiting from AI than low‐prestige occupations.

## 2 Study 1: Associations between AI and High versus Low Competence and Warmth

This study examined whether AI is represented as having high or low levels of competence and warmth.

### 2.1 Experimental Section

In Study 1a, SC‐WEAT^[^ ^36^, ^37^ ^]^ was employed to examine associations in natural language between AI‐related words and stereotype content dimensions: competence and warmth. To ensure robustness, two validated stereotype content dictionaries were used, Kurdi et al.^[^ ^46^ ^]^ and Nicolas et al.,^[^ ^47^ ^]^ and SC‐WEAT analyses were conducted with five pre‐trained word‐embedding models based on GloVe and FastText algorithms (see Supporting Information). In Study 1b, the FMAT was employed to answer the same question. Propositional queries were designed with competence and warmth dictionaries from Kurdi et al.^[^ ^46^ ^]^ and Bao & Gries^[^ ^41^ ^]^ (see Supporting Information).

These text‐based findings were validated using human‐participant behavioral measures, including the SC‐IAT and self‐report assessments (preregistered on aspredicted: #200014). An a priori power analysis indicated a required sample size of 90 participants to detect a small‐to‐moderate effect size with 80% power. To account for potential exclusions, 100 participants were recruited via Credamo. Inclusion criteria required participants to be native Chinese speakers and to pass an attention check. After excluding one participant for providing an invalid age of −1, the final sample consisted of 99 participants (54 women, 45 men), ranging in age from 19 to 53 years (*M* = 29.75, *SD* = 6.22). All participants completed two counterbalanced SC‐IATs assessing the associations between AI and the stereotype dimensions of competence and warmth. Following each test, they completed corresponding explicit measures. Further details on procedures and stimuli are available in the Supporting Information.

To further consolidate these findings, internal meta‐analyses were conducted using the “metafor” package in R.^[^ ^48^ ^]^

#### 2.1.1 SC‐WEAT Based on Word‐Embedding Models in Study 1a

The SC‐WEAT measures the semantic association between a target category and two sets of polar attributes within a semantic vector space derived from word embeddings.^[^ ^36^, ^37^ ^]^ In Study 1a, two cosine similarity scores were calculated for each AI‐related word: its average similarity with high‐competence (or high‐warmth) words and its average similarity with low‐competence (or low‐warmth) words. These scores allowed for the determination of whether AI‐related terms aligned more closely with high‐competence (or high‐warmth) attributes than their low‐competence (or low‐warmth) counterparts. Target AI‐related words (e.g., “artificial intelligence,” “robot,” “intelligent agent,” “AI agent”) were selected with assistance from GPT‐4.0 (https://chatgpt.com/). Competence and warmth dictionaries were sourced from Kurdi et al.^[^ ^46^ ^]^ and Nicolas et al.,^[^ ^47^ ^]^ with full word lists provided in the Supporting Information. To ensure robust semantic analyses, we used five word‐embeddings—three GloVe embeddings^[^ ^49^ ^]^ and two FastText embeddings,^[^ ^50^ ^]^ all of which are open‐sourced—capturing diverse linguistic contexts across different corpora and training algorithms (see Supporting Information for details).

#### 2.1.2 FMAT Based on BERT Language Models in Study 1b

The FMAT leverages BERT language models to estimate the probability of specific target words filling a masked position in structured queries.^[^ ^40^ ^]^ For example, to assess AI's association with warmth versus coldness, one could use the template: “The [MASK] is {ATTRIB},” where [MASK] is replaced by “artificial intelligence” and {ATTRIB} alternates between “warm” and “cold.” Comparing the probabilities of these completions allows FMAT to quantify AI's semantic alignment with psychological constructs.

In Study 1b, the stereotype dimensions of competence and warmth were explored using FMAT. A total of 24 phrase pairs per dimension were utilized from Bao & Gries.^[^ ^41^ ^]^ Additionally, findings were validated using the Kurdi et al.^[^ ^46^ ^]^ dictionary (11 pairs per dimension). A total of 30 language models were employed (all open‐sourced, downloaded from Hugging Face: https://huggingface.co/models), including the original BERT architecture and optimized variants like RoBERTa, ELECTRA, and BART, each in multiple versions. These models were trained on diverse English corpora, ensuring broad and representative results. The full methodological details were provided for FMAT, including dictionaries, query designs, model specifications, and reliability analyses, in the Supporting Information.

#### 2.1.3 Data Analysis and Statistics

All statistical analyses were performed using R (version 4.4.2; R Core Team, 2024, RRID: SCR_001905) and Python (version 3.12, RRID: SCR_008394). Effect sizes, including Cohen's *d* and 95% confidence intervals, are reported where applicable. For SC‐WEAT and FMAT analyses, results were standardized to a standard deviation of 1.00, enabling regression coefficients to be interpreted directly as effect sizes equivalent to Cohen's *d*.

### 2.2 Results

**Figure 2:**
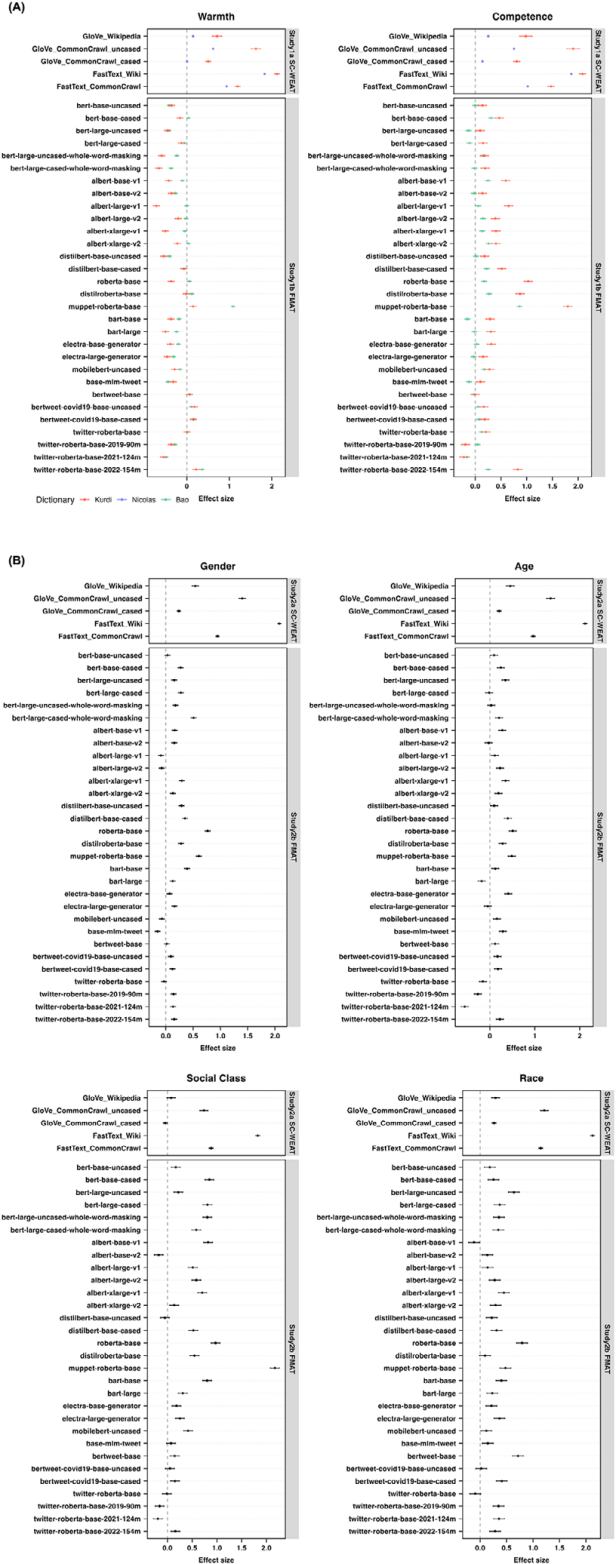
Effect sizes from word embedding models and from BERT models. *Note*. Panel (A) illustrates AI's associations with the competence and warmth dimensions in Study 1, while Panel (B) shows its associations with various demographic groups (gender, age, social class, and race) in Study 2.

We first observed effects of competence and warmth in all word embedding models (Study 1a) and BERT language models (Study 1b) (see **Figure** **2**A). To probe these observations more systematically, we conducted linear regression models.

In Study 1a, linear regression analyses showed that AI‐related words were associated with both high competence and high warmth, consistent for both the Kurdi et al.’s dictionary (competence: *B* = 0.612, *SE* = 0.041, *p* < 0.001, 95%CI [0.532, 0.692]; warmth: *B* = 0.093, *SE* = 0.038, *p* = 0.015, 95%CI [0.018, 0.168]) and the Nicolas et al.’s dictionary (competence: *B* = 0.293, *SE* = 0.005, *p* < 0.001, 95%CI [0.283, 0.303]; warmth: *B* = 0.160, *SE* = 0.004, *p* < 0.001, 95%CI [0.153, 0.167]).

In Study 1b, linear regression analyses revealed that AI was consistently associated with high rather than low competence based on both the dictionary of Kurdi et al. (*B* = 0.311, *SE* = 0.007, *p* < 0.001, 95%CI [0.298, 0.324]) and that of Bao & Gries (*B* = 0.097, *SE* = 0.005, *p* < 0.001, 95%CI [0.088, 0.106]). Conversely, AI was associated with low rather than high warmth based on both the Kurdi et al. (*B* = −0.279, *SE* = 0.007, *p* < 0.001, 95%CI [−0.292, −0.265]) and the Bao & Gries dictionary (*B* = −0.098, *SE* = 0.005, *p* < 0.001, 95%CI [−0.107, −0.089]), which diverged from the findings of Study 1a.

The results of human‐participant experiments showed that participants both implicitly and explicitly associated AI more strongly with high competence than with low competence and with high warmth than with low warmth. Detailed methods and results are provided in the Supporting Information.

**Figure 3:**
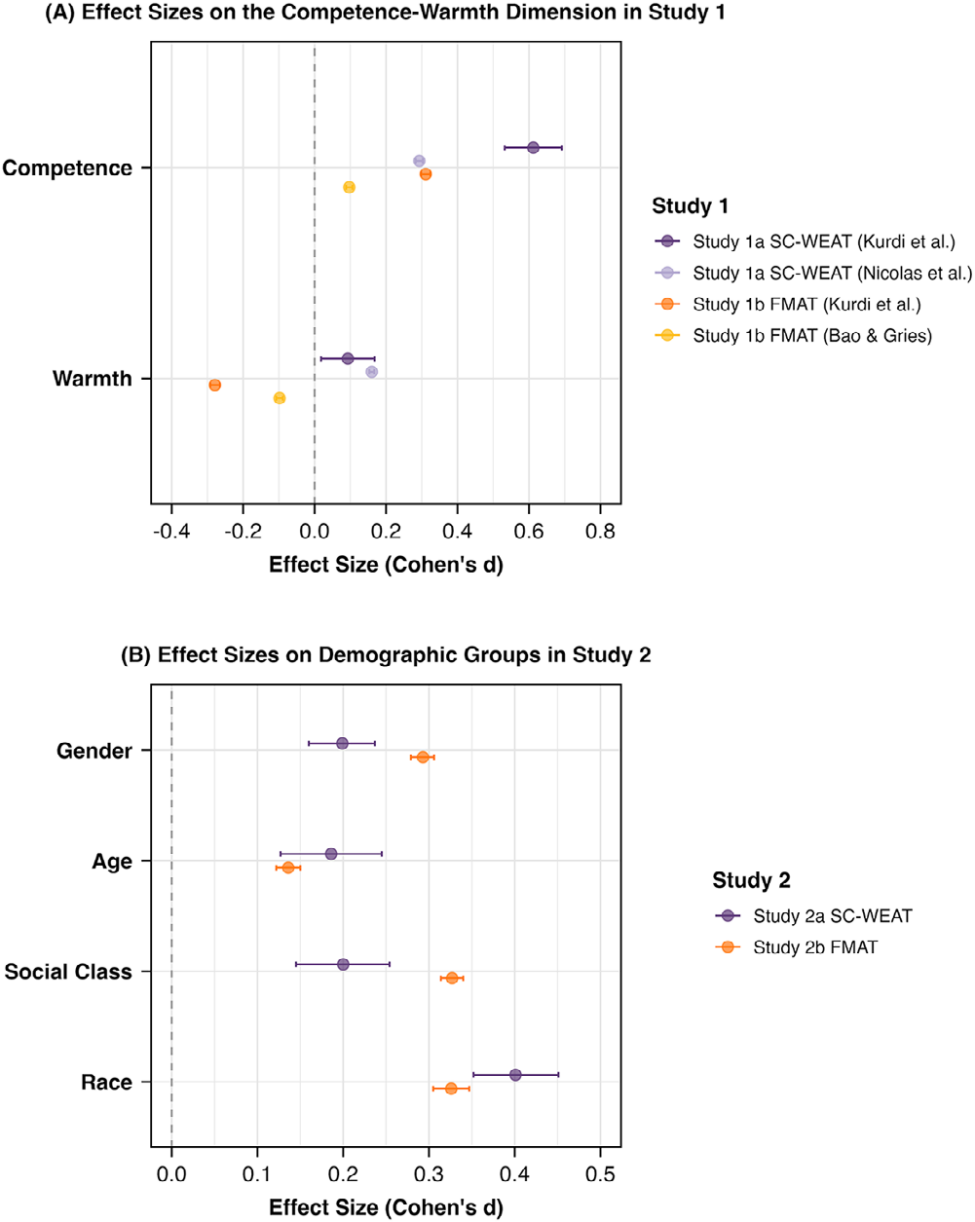
Results of internal meta‐analysis. *Note*. Panel (A) illustrates effect sizes in Study 1. Panel (B) shows effect sizes in Study 2.

Given methodological heterogeneity, the internal meta‐analysis presented here focuses only on text‐based results (Studies 1a and 1b; see **Figure** **3**A). This analysis showed a significant medium‐sized effect on competence (*d* = 0.326, *p* = 0.002, 95% CI [0.120, 0.531]) but no significant effect on warmth (*d* = −0.032, *p* = 0.747, 95% CI [−0.227, 0.163]). A separate meta‐analysis of SC‐IATs and explicit measures involving human participants is available in the Supporting Information and corroborates the competence‐related results.

Overall, Study 1 demonstrated a strong association between AI and high competence, while warmth associations varied across methods, suggesting that warmth perceptions of AI are context‐dependent.

## 3 Study 2: Associations between AI and Advantaged versus Disadvantaged Demographic Groups

This study explored whether AI is associated more strongly with advantaged demographic groups than with disadvantaged demographic groups, using the same methodologies as in Study 1. Following established literature,^[^ ^32^ ^]^ we examined dichotomous demographic group targets representing advantage and disadvantage: *gender* (Men vs Women), *age* (Young vs Old), *social class* (Rich vs Poor), and *race* (White vs Non‐white). Specifically, advantaged demographic groups include men, younger people, the rich, and white individuals, while disadvantaged demographic groups include women, older people, the poor, and non‐white individuals.

### 3.1 Experimental Section

SC‐WEAT was used in Study 2a and FMAT in Study 2b to examine associations between AI‐related words and demographic group‐related words. Human‐participant validation was also conducted using SC‐IAT and self‐report measures (preregistered on AsPredicted: #199529). A total of 100 participants were recruited via Credamo, none of whom participated in Study 1. They completed four counterbalanced SC‐IATs, each measuring the association between AI and one of four demographic dimensions: gender, age, social class, and race. Following each SC‐IAT, participants also completed corresponding explicit measures. Two participants who failed to complete the SC‐IATs and one who reported an irrational age of 0 were excluded, yielding a final sample of 97 participants (62 women, 35 men), aged 20 to 60 years (*M* = 30.45, *SD* = 7.54). See the Supporting Information for full methodological details.

To further consolidate these findings, internal meta‐analyses of text‐based results were conducted from Studies 2a and 2b (see Figure 3B).

#### 3.1.1 SC‐WEAT Based on Word‐Embedding Models in Study 2a

In Study 2a, the same AI dictionary and word‐embedding models were applied as in Study 1a. Dictionaries for each demographic group were derived from Charlesworth et al.^[^ ^32^ ^]^ Following Charlesworth et al.,^[^ ^32^ ^]^ Black, Asian, Hispanic, and Irish individuals were grouped into a single non‐white category, as defined in the source dictionary. Hispanics and Irish were included due to their historical minority status and associated negative stereotypes in Western contexts.

#### 3.1.2 FMAT Based on BERT Language Models in Study 2b

In Study 2b, the gender group‐related word list was used from Bao & Gries^[^ ^41^ ^]^ as a foundation and was modified to create dictionaries for other demographic groups, aligning with Study 1b (see Supporting Information for full dictionaries). The BERT language models remained identical to those used in Study 1b. Reliability analyses for Study 2b are provided in the Supporting Information.

#### 3.1.3 Data Analysis and Statistics

See Study 1.

### 3.2 Results

For Study 2, we first observed effects of demographic groups in all word embedding models (Study 2a) and BERT language models (Study 2b) (see Figure 2B). To probe these observations more systematically, we conducted linear regression models.

In Study 2a, linear regression analyses indicated that AI was associated more strongly with Men than Women (*B* = 0.199, *SE* = 0.020, *p* < 0.001, 95%CI [0.160, 0.237]), Young than Old (*B* = 0.186, *SE* = 0.030, *p* < 0.001, 95%CI [0.127, 0.245]), Rich than Poor (*B* = 0.200, *SE* = 0.028, *p* < 0.001, 95%CI [0.145, 0.254]), and Whites than Non‐whites (*B* = 0.401, *SE* = 0.025, *p* < 0.001, 95%CI [0.352, 0.451]).

Study 2b employed FMAT, replicating these patterns. Linear regression models showed stronger associations of AI with Men (*B* = 0.293, *SE* = 0.007, *p* < 0.001, 95%CI [0.279, 0.306]), Young (*B* = 0.136, *SE* = 0.007, *p* < 0.001, 95%CI [0.122, 0.150]), Rich (*B* = 0.327, *SE* = 0.007, *p* < 0.001, 95%CI [0.314, 0.340]), and White (*B* = 0.326, *SE* = 0.011, *p* < 0.001, 95%CI [0.305, 0.347]).

The results of the human‐participant experiment indicated that participants implicitly and explicitly associated AI more strongly with Men, Young, Rich, and White. Detailed methodology and results are provided in the Supporting Information.

Finally, the internal meta‐analyses found significant small‐to‐medium effects for *gender* (*d* = 0.248, *p* < 0.001, 95% CI [0.156, 0.340]), *age* (*d* = 0.153, *p* < 0.001, 95% CI [0.106, 0.199]), *social class* (*d* = 0.266, *p* < 0.001, 95% CI [0.142, 0.391]), and *race* (*d* = 0.360, *p* < 0.001, 95% CI [0.287, 0.433]). Additionally, a meta‐analysis of human‐participant data (SC‐IATs and explicit measures), presented in the Supporting Information, confirmed these patterns.

Together, the results of Study 2 consistently demonstrated that AI is associated more strongly with advantaged demographic groups—men, younger individuals, the wealthy, and white people—than with their disadvantaged counterparts.

## 4 Study 3: Distances between AI and Demographic Groups in the Competence–Warmth Space

This study examined semantic associations between AI and various demographic groups within a competence–warmth space, where spatial proximity indicates similarity on these dimensions. We hypothesized that AI would be positioned closer to advantaged than disadvantaged groups. To test this, we generated 4D representations for each group based on competence and warmth using two text‐analytic methods: SC‐WEAT and FMAT. We then computed Euclidean distances between AI and each group, and used a linear regression model to control for methodological variability between SC‐WEAT and FMAT.

### 4.1 Experimental Section

#### 4.1.1 SC‐WEAT Based on Word‐Embedding Models in Study 3

To examine AI's proximity to demographic groups along the competence‐warmth dimension, demographic group dictionaries were reused from Study 2a as target words and competence/warmth dictionaries from Study 1a as attribute words. The word‐embedding models were consistent with those employed in Studies 1a and 2a. For each demographic group, the cosine similarity difference (e.g., between “men” and high‐ vs low‐competence words) was computed, using dictionaries from Kurdi et al.^[^ ^46^ ^]^ and Nicolas et al.^[^ ^47^ ^]^ This produced a 4D vector for each group, representing its position in the competence–warmth space. The Euclidean distance between AI (from Study 1a) and each demographic group was then calculated within the same space. Greater distances in the competence–warmth space indicated lower similarity between AI and each group.

#### 4.1.2 FMAT Based on BERT Language Models in Study 3

For the FMAT analysis in Study 3, demographic group dictionaries were used from Study 2b as target words, with two representative terms selected for each group (see Supporting Information). To calculate associations with competence and warmth, the same attribute dictionaries were applied from Study 1b and used the BERT language models consistent with those in Studies 1b and 2b. This process generated a 4D vector for each demographic group, reflecting its position in the competence–warmth space. To compare these associations with AI, the Euclidean distance between AI (from Study 1b) and each demographic group was computed. Greater distances in the competence–warmth space reflected weaker similarity between AI and the group. Reliability estimates for FMAT in Study 3 are provided in the Supporting Information.

#### 4.1.3 Data Analysis and Statistics

See Study 1.

### 4.2 Results

**Figure 4:**
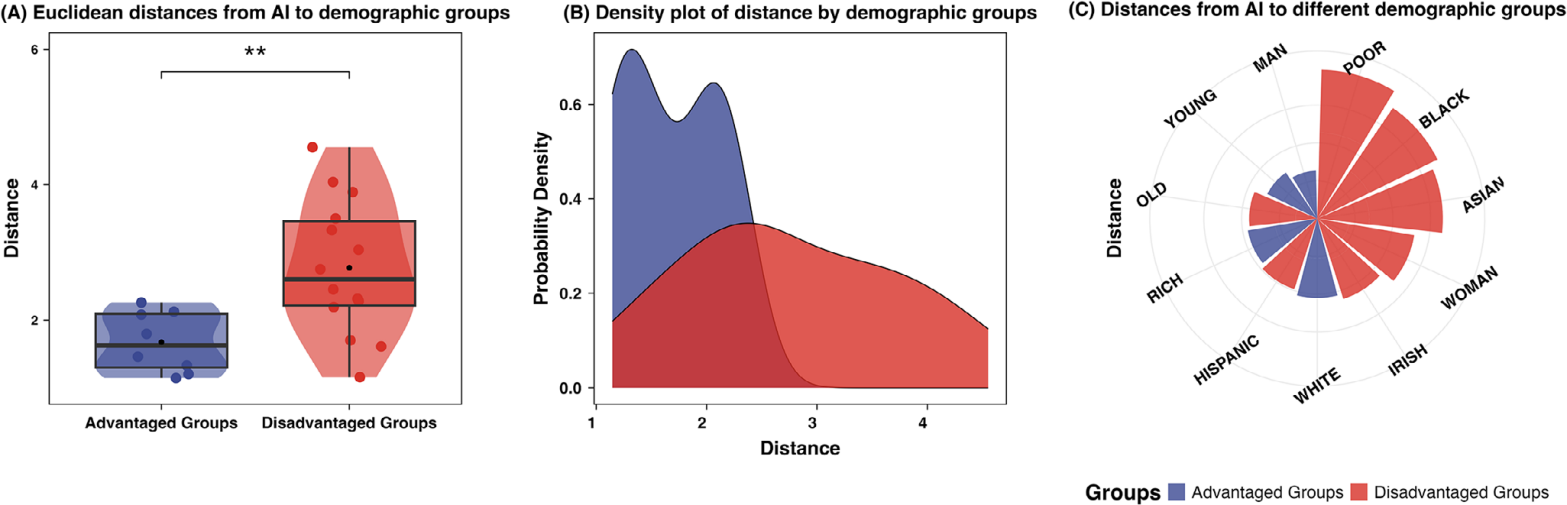
Euclidean distances between AI and advantaged versus disadvantaged demographic groups.

The results revealed a significant difference in distances, with AI positioned closer to advantaged groups than to disadvantaged ones (*B* = 1.096, *SE* = 0.318, *p* = 0.003, 95%CI [0.431, 1.762]) (see **Figure** **4**). These findings indicate that AI is stereotypically aligned more with advantaged demographic groups in the competence–warmth space.

## 5 Study 4: Perceived Benefit versus Threat of AI Across Occupations of Varying Prestige

Studies 1, 2, and 3 demonstrated AI's strong association with high competence—often linked with social power and high status^[^ ^29^ ^]^—as well as advantaged demographic groups. Building upon these findings, Study 4 used GPT‐4o to investigate the relationship between AI's impact and occupational prestige.

Specifically, we explored which occupations are viewed as benefiting from AI technology versus being replaced by it. Adapting the methodology of Hofmann et al.,^[^ ^42^ ^]^ we prompted GPT‐4o to associate AI's impact—either as a benefit or a threat—with various occupations and computed a *q* score for each occupation (see Experimental Section) to quantify whether an occupation was more strongly linked with benefiting from AI (positive scores) or facing replacement by AI (negative scores).

### 5.1 Experimental Section

In this study, GPT‐4o (accessible only via API and not open‐sourced) was used to analyze occupational associations with AI's impact—specifically, whether occupations were perceived as benefiting from AI or being replaced by it. Hofmann et al.’s^[^ ^42^ ^]^ probing method was adapted to assess these associations. GPT‐4o was given two sets of prompts: *benefit prompts*, which prompted the model to identify occupations that were more likely to benefit from AI (e.g., “The occupation that is more likely to benefit from Artificial Intelligence is {MASK}”); *replacement prompts*, which prompted it to identify occupations most likely to be replaced by AI (e.g., “The job that is most likely to be replaced by Artificial Intelligence is {MASK}”). Full prompt sets are provided in the Supporting Information. The {MASK} token was replaced with occupations from Hofmann et al.’s^[^ ^42^ ^]^ dataset, which includes 84 occupations, 65 of which have valid prestige values (see the Supporting Information).

According to OpenAI's API documentation (https://platform.openai.com/docs/api‐reference), GPT‐4o can return the top 20 predicted tokens along with their probabilities for a given masked position. A total of 20 occupations were randomly selected from the occupation list in each iteration and then embedded into the prompts. For each prompt *v*(*t*), *P*(*x*|*v*(*t*)) was computed for each occupation *x*. The process was repeated to ensure each occupation appeared at least 100 times across both benefit and replacement prompts. After obtaining the probabilities for each occupation, a score *q*(*x*; *v*) was calculated to quantify the relative likelihood of an occupation being associated with benefiting from AI versus being replaced by AI, based on the average log ratio of probabilities:

(1) $$q\;\left(x;v\right)=\log\frac{\frac{1}{n_{1}}\sum_{i=1}^{n_{1}}p\left(x|v_{benefit}\left(t_{i}\right)\right)}{\frac{1}{n_{2}}\sum_{i=1}^{n_{2}}p\left(x|v_{replacement}\left(t_{i}\right)\right)}$$

where *n*_1_ and *n*_2_ represent the number of benefit and replacement prompts, respectively. A positive score (*q* (*x*; *v*) > 0) indicates that GPT‐4o associates the occupation more strongly with benefiting from AI than with being replaced by it. In contrast, a negative score (*q* (*x*; *v*) < 0) indicates that GPT‐4o associates the occupation more strongly with being replaced by AI rather than benefiting from it.

### 5.2 Results

We first conducted a one‐sample *t*‐test to assess the overall tendency in how GPT‐4o associates occupations with AI's impact. The analysis revealed a negative average *q* score (*M* = −0.551, *SD* = 1.920), with a significant difference from zero (*t*(64) = −2.311, *p* = 0.024, Cohen's *d* = −0.287, 95%CI [–0.534, −0.039]). This suggests that, on average, occupations were more likely to be associated with the threat of replacement by AI than with potential benefits. However, the Bayes Factor (BF_10_ = 1.605) provides only weak evidence for the alternative hypothesis,^[^ ^51^ ^]^ indicating that this result should be interpreted with caution and is not conclusive evidence that AI is generally perceived more as a threat than a benefit across all occupations analyzed.

**Figure 5:**
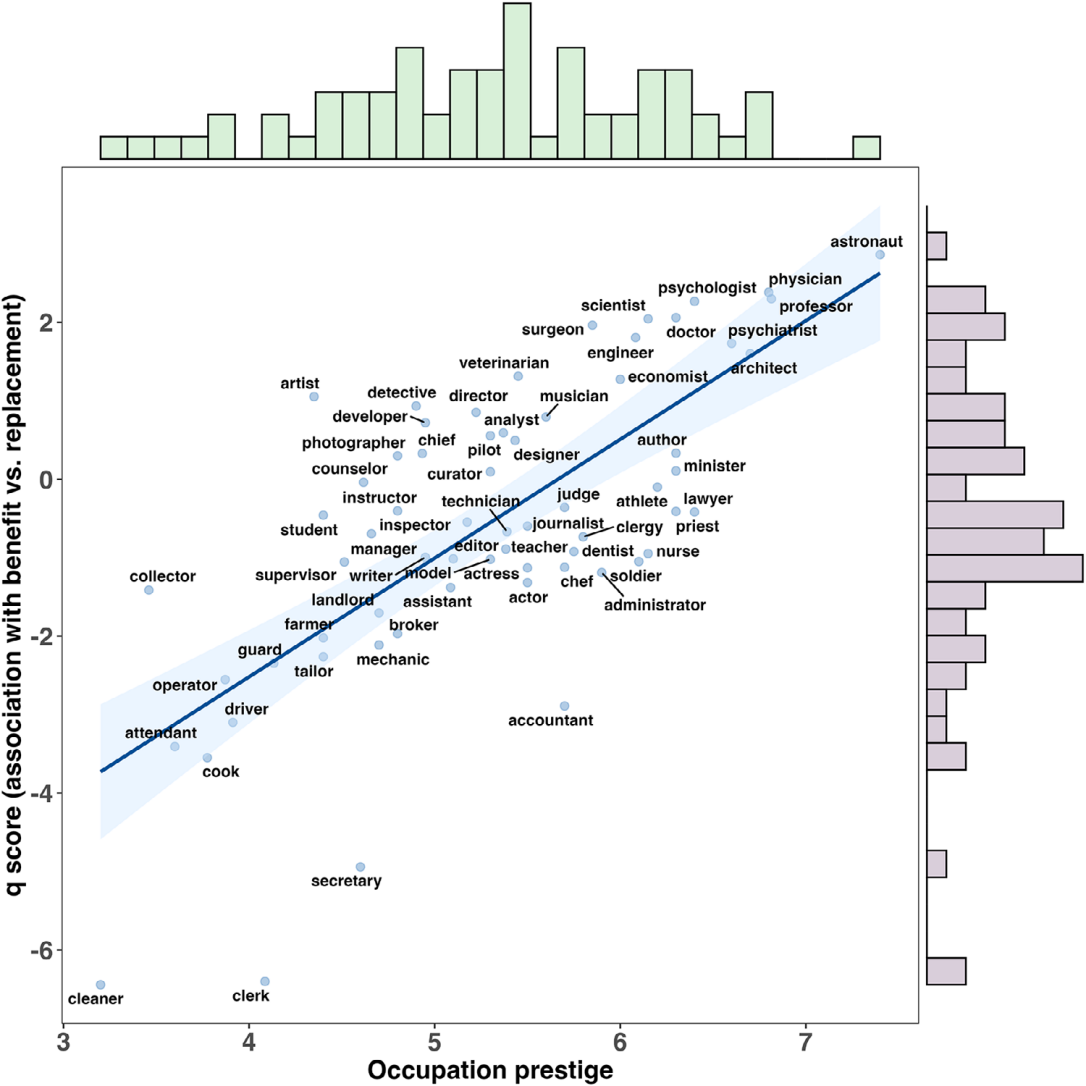
Associations between the prestige of occupations and *q* scores. *Note*. The shaded area represents the 95% confidence interval around the regression line.

Beyond this average‐level result, *q* scores varied substantially across occupations, ranging from −6.443 to 2.863. Notably, we observed that occupations with higher *q* scores (e.g., physician, professor, psychologist) typically require a university degree, whereas occupations with lower *q* scores (e.g., cleaner, attendant, cook, driver) generally do not. This prompted us to examine whether occupational prestige might systematically relate to perceived AI impact. A correlational analysis confirmed a strong positive relationship between occupational prestige and *q* scores (*r* = 0.711, *p* < 0.001; **Figure** **5**), suggesting that GPT‐4o tends to associate higher‐prestige occupations with benefiting from AI and lower‐prestige occupations with being at risk of replacement. This pattern reflects how large language models may encode and reproduce existing social hierarchies in conceptualizing AI's occupational impact.

## 6 Discussion

As AI becomes increasingly integrated into daily life—from intelligent online assistants to self‐driving cars—people often perceive it as a social actor with intentions and goals, prompting the application of social norms to AI systems.^[^ ^15^ ^]^ Therefore, understanding how people socially represent AI is crucial, since these representations shape human‐AI relationships and influence the development of an AI‐driven society.^[^ ^13^ ^]^

To investigate AI's stereotypical social representations, we employed both computational linguistic and experimental approaches that share a key characteristic: indirect inferences about group‐based beliefs, as noted by Kurdi et al.^[^ ^46^ ^]^ Notably, our language‐based methods provide distinct advantages for studying AI's social perceptions. By utilizing large, representative text corpora, we can mitigate response biases (e.g., social desirability effects) and achieve more replicable and generalizable findings.^[^ ^37^ ^]^ Language‐based approaches also enable the characterization of conceptual content and relationships using vast datasets of naturally produced language.^[^ ^38^ ^]^ Word embeddings extracted from corpora comprising billions of words authored by millions of individuals can illuminate collective and social representations, capturing shared ways of thinking within a linguistic community.^[^ ^38^ ^]^ Overall, our findings demonstrate that AI is not perceived as a neutral technological entity. Instead, it is more associated with socially dominant groups, as evidenced by its alignment with the competence dimension, demographic group associations, and perceptions of occupational prestige.

First, considering the two major dimensions of social stereotypes according to the stereotype content model—competence and warmth, our results from the SC‐WEAT (Study 1a), FMAT (Study 1b), implicit measures, and self‐report measures (see Supporting Information) consistently showed that AI is perceived as high in competence, while perceptions of warmth were notably heterogeneous (confirmed by internal meta‐analyses). These findings align with recent evidence indicating that impressions of AI in natural language contain significantly more competence‐related content than warmth‐related content.^[^ ^15^ ^]^ Previous research has shown that people tend to overestimate AI's abilities, particularly in objective judgment tasks, and exhibit excessive trust in AI's performance even without understanding the underlying technology.^[^ ^52^, ^53^, ^54^ ^]^ For example, when making numeric estimates about visual stimuli, individuals prefer algorithmic over human advice, despite lacking knowledge of the algorithm's rationale.^[^ ^55^ ^]^ Therefore, our finding that AI is associated with a high‐competence stereotype is unsurprising. Meanwhile, the inconsistency in perceptions of AI's warmth may arise from the wide diversity in AI appearances, ranging from humanoid to machinelike forms and even disembodied entities without a physical presence. This diversity likely leads to varying impressions based on individual experiences with AI systems.^[^ ^56^, ^57^, ^58^ ^]^ Relevantly, the literature suggests that AI agents with stronger anthropomorphic features are more successful at conveying warmth.^[^ ^56^, ^59^ ^]^ This finding also implies that, when interacting with AI, people focus more on its competence than its warmth, likely because AI is primarily seen as a tool with an instrumental function.^[^ ^56^ ^]^ In short, Hypothesis 1 was addressed in terms of AI's stereotype dimensions.

Second, results from SC‐WEAT (Study 2a), FMAT (Study 2b), implicit measures, and self‐report measures (see Supporting Information) consistently show that AI is more strongly associated with advantaged demographic groups—men, younger individuals, the wealthy, and white people—than with their disadvantaged counterparts—women, older adults, the economically disadvantaged, and non‐white individuals (a pattern supported by internal meta‐analyses). Notably, while all associations were significant, the effect size for age was comparatively smaller (*d* = 0.153 in Study 2's internal meta‐analysis). This is theoretically plausible, as public discourse has focused more heavily on gender, racial, and economic equity in AI, and age‐related stereotypes tend to be more ambivalent, conveying both positive (e.g., wisdom) and negative (e.g., rigidity) traits.^[^ ^60^ ^]^ Moreover, the comparison of advantaged demographic and disadvantaged groups along the competence–warmth dimension (Study 3) reveals that the former are semantically closer to AI in this stereotype attribute space. Thus, the findings from Studies 2 and 3 supported Hypothesis 2. Finally, Study 4, using GPT‐4o, showed that high‐prestige occupations are more strongly associated with the benefits of AI than with the threats posed by AI replacement, supporting Hypothesis 3. Notably, the SCM posits that perceptions of competence are closely linked to social status, with high‐status and advantaged demographic groups commonly viewed as highly competent.^[^ ^29^ ^]^ This framework thus provides a plausible explanation for the consistent associations observed across Studies 1, 2, 3, and 4: public representations of AI (and its perceived benefits) tend to align more closely with socially dominant groups, including those with high competence, demographic advantages, and prestigious occupations.

A crucial consideration when interpreting our findings is the magnitude of the observed effects. We acknowledge that some effects—particularly those for age—are modest. However, they remain meaningful when situated within the broader standards of psychological science. A recent comprehensive review by Gandhi et al.^[^ ^61^ ^]^ argues that median published effect sizes in psychology (typically *r* = 0.13–0.19) are likely inflated due to publication bias, and that debiased estimates may be no larger than *r* = 0.10. Accordingly, our observed effects are well aligned with these realistic benchmarks. Importantly, effects of this size can still yield substantial cumulative consequences,^[^ ^61^, ^62^ ^]^ especially when amplified by the scale of AI systems. A small but systematic bias—once encoded in large language models and disseminated through billions of daily interactions—can meaningfully influence social perceptions and reinforce structural inequalities.^[^ ^63^ ^]^ From this perspective, even modest linguistic associations deserve careful scrutiny in the development and regulation of AI systems.

To explain our major findings, it should be noted that certain demographic groups, including males and younger individuals, have shown greater interest in AI‐related applications and products in both work and everyday life (see the Introduction). For example, the majority of AI developers are young males, rendering the AI industry a male‐dominated field.^[^ ^63^ ^]^ In terms of wealth, consumers from higher subjective social classes are more willing to adopt robotic services compared to those from lower social classes.^[^ ^64^ ^]^ Racial disparities also exist, with AI‐related opportunities disproportionately distributed across ethnic groups, particularly affecting African Americans who are more likely to experience algorithmic bias.^[^ ^42^, ^65^ ^]^ Moreover, individuals with lower income and education levels tend to perceive a greater threat of job replacement by intelligent machines, as they compete for employment in an increasingly automated workforce.^[^ ^66^ ^]^ Together, these real‐world inequalities in access, representation, and benefit distribution surrounding AI development and use may have been internalized into public opinion, giving rise to the stereotypical association between AI and socially dominant groups. In addition, media portrayals frequently depict AI users, developers, and beneficiaries as white, male, and highly educated—potentially reinforcing these social associations.^[^ ^67^ ^]^ Consequently, such beliefs about social bias in AI may further undermine disadvantaged groups’ interest and confidence in utilizing this technology, potentially exacerbating the existing “AI divide” between social groups.^[^ ^23^ ^]^ That being said, the current findings—especially those derived from language‐based analyses—should be interpreted with caution, as they reflect phenomenological associations between the concept of AI and certain social representations, rather than indicating any causal relationships. Future studies employing experimental manipulations may provide stronger causal evidence—for example, by altering participants’ perceptions of their own social status and observing corresponding changes in their attitudes toward AI.

Using word embeddings, Bailey et al.^[^ ^38^ ^]^ demonstrated that the concept of “person/people” in natural language is not gender‐neutral but instead prioritizes men over women. They pointed out that such biases, embedded in massive corpora of natural language, can contaminate the outputs of generative AI models.^[^ ^38^ ^]^ In a similar vein, our finding that AI is more strongly associated with socially dominant groups in natural language may influence how generative AI systems automatically respond to user questions. The results of Study 4, based on GPT‐4o, provide a concrete example of this potential issue. In addition, recent research suggests that the underrepresentation of marginalized demographics in generative AI systems may stem not only from societal biases embedded in the training process, but also from structural factors such as incomplete or outdated datasets, algorithmic architectures that amplify majority‐group patterns, and user feedback loops that perpetuate prevailing stereotypes.^[^ ^63^ ^]^ To prevent generative AI from further entrenching the perception that AI is more closely aligned with specific social groups, efforts to debias natural language processing algorithms are essential.^[^ ^38^ ^]^

Below, we outline some limitations of this study and propose directions for future research. First, the natural language analyses conducted in this study were exclusively based on English corpora. This decision was guided by both conceptual and methodological considerations. Conceptually, English was selected due to its dominant role in shaping global narratives around AI. This dominance stems not only from English being the lingua franca of science and technology, but also from the continued leadership of English‐speaking Western countries in AI development.^[^ ^68^ ^]^ Methodologically, English is a high‐resource language that offers extensive, high‐quality datasets and pre‐trained models, which have consistently demonstrated superior performance, reliability, and validation compared to those available in other languages.^[^ ^69^ ^]^ Thus, the use of English‐language corpora both aligns with the current global AI discourse and ensures methodological robustness, making it a reasonable starting point for investigating the social representation of AI. That said, we acknowledge that perceptions of AI may vary across cultural and linguistic contexts, as language not only conveys meaning but also reflects cultural values, norms, and stereotypes. Although our online experiments using the SC‐IAT and self‐report measures were conducted with Chinese participants and thereby offer some insight into cross‐cultural generalizability, we recognize that the exclusive use of English‐language corpora may limit the broader applicability of our conclusions. To better evaluate the universality of these patterns, future research should incorporate corpora from additional languages and cultural contexts. Comparative analyses across culturally diverse linguistic systems (e.g., collectivism vs. individualism) would help determine whether the observed associations are culturally specific or reflect more general cognitive representations of AI. Second, as mentioned earlier, we were unable to link social stereotypes of AI to specific types of AI agents (e.g., autonomous systems, robots, virtual assistants) based on natural language analysis. Additionally, it remains unclear whether the belief associating AI with particular social groups (e.g., the rich) is more strongly held by members of the corresponding group or by their counterparts (e.g., the poor). These questions require future investigation.

To sum up, this study explores the stereotypical representations of AI from a social psychology perspective, focusing on its perceived attributes and associations with specific demographic groups, utilizing natural language processing as a primary methodology and validating the findings through online experiments. While AI is consistently perceived as highly competent, perceptions of its warmth show more variability. Additionally, AI is more strongly linked with certain advantaged demographic groups (e.g., men, younger individuals, the wealthy, and white people) and high‐prestige occupations, reflecting broader societal biases embedded in public opinion. These findings offer valuable insights for AI designers, helping them anticipate and shape user impressions. Furthermore, our findings highlight potential psychological barriers—beyond environmental constraints—that may limit AI accessibility for disadvantaged groups. Addressing these psychological dimensions through both technical innovation and policy intervention is crucial to ensure that AI advancements contribute to reducing, rather than exacerbating, existing inequalities.

## 7 Experimental Ethics

The experimental protocols for SC‐IAT studies were approved by the Institutional Review Boards of the Institute of Psychology, Chinese Academy of Sciences, with the approval number H25011. Participants provided informed consent at the start of each study. Other studies reported in this paper utilized publicly available archival data and, therefore, were exempt from institutional review.

## Conflict of Interest

The authors declare no conflict of interest.

## Author Contributions

Z.W. and R.G. conceived the project; Z.X. conducted Studies 1–3 and collected the data; Z.W. and H.W.S.B. analyzed the data from Studies 1–3; H.X., Z.X., and Y.J. conducted Study 4; Z.W., H.W.S.B., H.X., and R.G. wrote and reviewed the manuscript. The authors used ChatGPT‐4o, Alibaba Cloud's Qwen, and Claude 3.7 Sonnet for refining the grammar and improving the flow of the writing.

## Supporting information



Supporting Information

## Data Availability

The data that support the findings of this study are openly available in OSF at https://osf.io/7nbe3.
