# Supplementary material for: Artificial Intelligence Is Stereotypically Linked More with Socially Dominant Groups in Natural Language
Source: Adv Sci (Weinh). 2025 Jul 28;12(39):e08623. doi: 10.1002/advs.202508623 (PMC12533212; doi:10.1002/advs.202508623)
Supplement: Supplementary file 1 — Supporting Information [file ADVS-12-e08623-s001.docx]

Supplementary Materials for

**Artificial Intelligence is Stereotypically Linked More with Socially Dominant Groups in Natural Language**

**This file includes:**

Supplementary Text

Tables S1 to S15

Figures. S1 to S8

References

Supplementary Text

# Study 1

## Study 1a

### *Dictionaries*

**Dictionary for Artificial Intelligence (AI)**

The AI-related dictionary was generated by the research team (Table S1).

**Dictionary for Competence and Warmth**

We used two established dictionaries for the Single-Category Word Embedding Association Test (SC-WEAT) in Study 1a: one developed by Kurdi et al. ^[1]^ and the other by Nicolas et al. ^[2]^

Kurdi et al.’s word list consisted of 11 words for high-warmth, 11 words for low-warmth, 11 words for high-competence, and 11 words for low-competence (Table S2). To ensure compatibility with case-sensitive embedding models, which distinguish between lowercase and capitalized versions of words, we expanded the dictionary by capitalizing the first letter of each word. This resulted in a final dictionary containing 22 words for each dimension.

Nicolas et al.’s word list was larger and more comprehensive. We accessed their competence–warmth (high vs. low) dictionaries via the R package *Semi-Automated Dictionary Creation* (SADCAT; https://github.com/gandalfnicolas/SADCAT). Similar to the Kurdi dictionary, we also expanded Nicolas et al.’s dictionary by capitalizing the first letter of each word. Additionally, given the need to compare competence and warmth between specific pairs of categories (e.g., high competence vs. low competence, high warmth vs. low warmth), we performed a de-duplication process to ensure that each word in the list was unique within its respective category. This step was crucial to eliminate any overlap or redundancy, which could otherwise distort the comparisons. The final Nicolas et al. dictionary contained 1,861 words for high-warmth, 4,293 words for low-warmth, 2,102 words for high-competence, and 1,180 words for low-competence (see Table S2 for example words; for a full list see online repository).

The combination of these two dictionaries enabled us to obtain robust results. While Kurdi et al.’s dictionary contains fewer words, it provides a more balanced comparison of high vs. low warmth and high vs. low competence within each dimension and effectively represents the core concepts of each. In contrast, although the dimensions in Nicolas et al.’s dictionary are less balanced, the larger number of words provides a more extensive coverage of the categories, allowing for a more nuanced analysis. The use of both dictionaries thus enhanced the reliability and depth of our findings.

***Supplementary Results: Multidimensional Scaling analysis***

In this supplementary analysis, we employed Multidimensional Scaling (MDS) to investigate the spatial relationships between AI and warmth-competence attributes. While the main analysis in Study 1a focuses on the distinctions between AI and high versus low warmth and competence, the MDS approach offers a complementary visualization by preserving the distance relationships between words in high-dimensional space, providing additional insights into these associations.

We utilized five distinct word embedding models (see Table S3), consistent with those outlined in Study 1a. These models were processed to obtain word embeddings for sets of words related to AI, as well as the warmth and competence attributes (the same dictionary used in Study 1a). For the MDS analysis, we averaged the word embeddings for each attribute category (warmth and competence) across the corresponding keywords, generating a representative vector for each attribute category. Next, we calculated the Euclidean distance between these vectors and applied MDS to reduce the dimensionality to three dimensions, thereby preserving the spatial relationships between the word groups as accurately as possible.

The snapshot of the 3D plot (Figure S1) illustrates the MDS results. In the competence dimension (Panel A), AI is positioned closer to high competence than to low competence. In the warmth dimension (Panel B), AI is similarly closer to high warmth than to low warmth. These visualizations provide insights into how AI is spatially positioned relative to warmth and competence attributes. Additionally, we have provided the interactive plot on OSF for further exploration (“supplementary_MDS analysis for study1a_2a/Study 1a MDS plot.html”).

**Study 1b**

***Method Details of Fill-Mask Association Test (FMAT)***

We followed Bao’s ^[3]^ workflow.

**Step 1:** We employed 30 BERT models from Hugging Face (<https://huggingface.co/models>) (see Table S4). These models were trained on diverse English corpora, including general-purpose datasets (e.g., Wikipedia, BookCorpus, and CommonCrawl) and domain-specific datasets (e.g., Twitter, Reddit, and specialized corpora on movies). All models were used to calculate the semantic probabilities of masked words in FMAT queries. The use of multiple models enhanced the robustness and generalizability of findings by mitigating potential biases from relying on a single model.

**Step 2:** FMAT queries provided linguistic contexts for models to estimate the likelihood of specific target words replacing the mask token. Queries were designed to be simple, clear, and to account for both singular and plural forms of target words. To ensure reliability, nine query templates were used (see Table S5). The [MASK] token was left blank, allowing models to compute semantic probabilities for AI-related words. The AI-related dictionary used in Study 1b is identical to that used in Study 1a. For Study 1b, {ATTRIB} was replaced with high and low pairs within each warmth and competence dimension (e.g., “competent” vs. “incompetent”) (see Table S6 for details).

**Step 3:** We computed the log probability ratio (LPR) for each target word $w$ between two phrases ($A$ vs. $B$) in each attribute pair:

$$\mathrm{LPR}\left( w \right)=\log\frac{P(w|\mathrm{attrib}_{A})}{P(w|\mathrm{attrib}_{B})}=\log P(w|\mathrm{attrib}_{A})-\log P(w|\mathrm{attrib}_{B})$$

The LPR index, which is normally distributed and less influenced by word frequencies, is more suitable for linear modeling than raw probabilities. FMAT analyses were conducted using all 30 BERT-based language models.

***Supplementary Results: Reliability Analysis and Results of Linear Mixed Modeling (LMM)***

**Reliability Analysis**

Besides testing the main results, we analyzed the reliability of our findings in three ways. First, to assess the inter-rater agreement among the 30 BERT-based models (treated as “raters”) in understanding the queries and estimating the probabilities (log-transformed), we computed the average-score intraclass correlation coefficient (ICC_average_), with both BERT language models and specific query sentences considered as random effects. For all query sentences, ICC_average_ = 0.841; for all query templates (i.e. nine queries), the ICCs were also high, ranging from 0.791 to 0.922 (Figure S2).

Second, we calculated the internal consistency of LPRs across query templates in both singular (*k* = 4) and plural (*k* = 5) forms, separately for each attribute dimension. As shown in Table S7, the results indicated good internal consistency among the queries (α_query (singular forms)_ = 0.852–0.880; α_query (plural forms)_ = 0.883–0.906).

Third, to assess the consistency of results across different query forms, we analyzed the simple effects within each query form. Specifically, we modeled LPR as a function of the three-way interaction between attributes, query forms, and models using the following formula in R (package “stats”): LPR ~ attribute * query form * model. Table S7 and Figure S5 summarize the resulting effect sizes (*d*), with 95% confidence intervals (CI).

**Results of LMM**

Considering the nest structure of data, we applied LMM using the “nlme” package in R. ^[4]^ The models treated the 30 BERT language models as random intercepts, meeting the Level-2 cluster size minimum of 10 as recommended by Snijders & Bosker. ^[5]^ The results of LMMs indicated that AI-related words were associated with high competence (Kurdi et al.: *B* = 0.311, *SE* = 0.063, *p* < 0.001, 95%CI [0.182, 0.440]; Bao & Gries: *B* = 0.097, *SE* = 0.037, *p* = .015, 95%CI [0.021, 0.173]) and low warmth (Kurdi et al.: *B* = –0.279, *SE* = 0.048, *p* < 0.001, 95%CI [–0.377, –0.180]; Bao & Gries: *B* = –0.098, *SE* = 0.061, *p* = 0.118, 95%CI [–0.222, 0.026]).

# Study 2

**Study 2a**

***Dictionaries***

**Dictionary for AI.**

The AI-related dictionary was identical to those used in Study 1a (Table S1).

**Dictionary for Demographic Groups**

We used the same dictionaries for each demographic group as derived from Charlesworth et al. ^[6]^ (Table S8). As in the analytic strategy in Study 1a, we expanded the dictionary by capitalizing the first letter of each word to ensure compatibility with case-sensitive embedding models.

***Supplementary results: Multidimensional Scaling analysis***

Identical to the supplementary analysis in Study 1a, we employed MDS to investigate the spatial relationships between AI and each demographic group. This analysis provides a visualization that preserves the distance relationships between words in high-dimensional space, offering additional insights into the associations between AI and advantaged demographic versus disadvantaged groups.

The word embedding models and analytic strategy were same as those in Study 1a. We obtained word vectors for AI and demographic groups (the same dictionary used in Study 2a). Then we calculated the Euclidean distance between these vectors and applied MDS to reduce the dimensionality to three dimensions.

The snapshot of the 3D plot (Figure S4) illustrates the MDS results. In terms of gender, age, and social class groups, the position of AI in space is closer to advantaged demographic groups compared to their counterparts—specifically, AI is closer to Man, Young, and Rich. Regarding racial groups, AI is closest to Asian, followed by White. This positioning may be influenced by stereotypes associating Asian individuals with strength in STEM fields. ^[7]^ Additionally, we have provided the interactive plots and versions that retain word embedding models’ information on OSF for further exploration (“supplementary_MDS analysis for study1a_2a/Study 2a MDS plot.html”).

**Study 2b**

***Method Details of FMAT***

As in Study 1b, we followed Bao’s ^[3]^ workflow and used the same 30 BERT models as in Study 1b. For Study 2b, we used nine query templates, four in singular form and five in plural form (see Table S9). As in Study 1b, the [MASK] token was used to have models compute the semantic probabilities for AI-related words, and the AI-related dictionary in Study 2b is identical to that used in Study 1b. Additionally, {ATTRIB} was replaced with socially advantaged and disadvantaged pairs within each demographic group (e.g., “masculine” vs. “feminine”; see Table S10).

***Supplementary Results: Reliability Analysis and Results of LMM***

**Reliability Analysis**

We also analyzed the reliability of the findings. First, to assess the inter-rater agreement among the 30 BERT language models (treated as “raters”), we computed the average-score intraclass correlation coefficient (ICC_average_). For all query sentences, ICC_average_ = 0.842; for all query templates in singular form (i.e. four queries), the ICCs were also high, ranging from 0.830 to 0.900, and for all query templates in plural form (i.e. five queries), the ICCs were also high, ranging from 0.804 to 0.914 (Figure S5).

Second, we calculated the internal consistency of LPRs across query templates in both singular (*k* = 4) and plural (*k* = 5) forms, separately for each demographic group. As shown in Table S11, the results indicated good internal consistency among the queries (α_query (singular forms)_ = 0.759–0.868; α_query (plural forms)_ = 0.861–0.903).

Third, to assess the consistency of results across different query forms, we analyzed the simple effects within each query form. Specifically, we modeled LPR as a function of the three-way interaction between attributes, query forms, and models using the following formula in R (package “stats”): LPR ~ attribute * query form * model. Table S11 and Figure S6 summarize the resulting effect sizes (*d*), with 95% CI.

**Results of LMM**

We applied LMM using the “nlme” package in R. ^[4]^ The results of LMMs indicated that AI-related words were associated with Man (*B* = 0.293, *SE* = 0.060, *p* < 0.001, 95%CI [0.169, 0.416]), Young (*B* = 0.136, *SE* = 0.040, *p* < 0.001, 95%CI [0.055, 0.218]), Rich (*B* = 0.327, *SE* = 0.070, *p* < 0.001, 95%CI [0.183, 0.471]) and White (*B* = 0.326, *SE* = 0.043, *p* < 0.001, 95%CI [0.238, 0.413]).

**Supplementary Studies 1 and 2: Evidence from Human-Participant Experiments**

Our validation was achieved using SC-IATs and their corresponding self-report measures. The SC-IAT follows the basic structure of the IAT but assesses the strength of associations for a single object (or category) by measuring reaction times (RTs) when the target is paired with two alternating attributes; shorter RTs in one pairing indicate stronger implicit associations. ^[8]^ We conducted two preregistered studies for validation respectively, one (AsPredicted: #200014; https://aspredicted.org/mhhv-zf4f.pdf) for Study 1, and the other (AsPredicted: #199529; https://aspredicted.org/p7y4-3b72.pdf) for Study 2.

**Methods**

***Participants and Statistical Power***

For the two SC-IAT studies (one is warmth-competence SC-IAT study, and one is demographic group SC-IAT study), priori power analyses were conducted using G*Power (Version 3.1.7, RRID: SCR_013726). ^[9]^ Based on previous SC-IAT research ^[8]^ we assumed a small to moderate effect size (*d* = 0.3). The analysis determined a required sample size of 90 participants to achieve sufficient power (80%) at $\alpha$ = 0.05. To account for potential dropouts or experimental errors, we recruited 100 participants per study via the Credamo platform (https://www.credamo.com/). Each participant was involved in only one study.

***Implicit Measures***

The competence–warmth SC-IAT study and demographic group SC-IAT study share the same procedure, with the distinction that the competence–warmth SC-IAT study consist of two SC-IATs, while the demographic group SC-IAT study include four SC-IATs (see Table S12).

In each study, participants were first directed to an introductory page outlining the experiment and their rights, where they provided demographic information, including gender, age, education level, and subjective social status. In the formal experiment, explicit measures were presented prior to implicit measures, as research suggests this order does not affect the psychometric properties or intercorrelation of these measures. ^[10]^

The SC-IAT measured the conceptual compatibility between the target (AI) and two attributes (e.g., “smart” and “dumb”) by comparing participants’ average response latencies across sorting tasks. The target words in both validation studies included “artificial intelligence,” “robot,” “intelligent agent,” “intelligent robot,” and “AI,” selected as core representations of AI. In the competence–warmth SC-IAT study, participants completed two SC-IATs assessing the stereotype dimensions of competence and warmth. The two SC-IATs were counterbalanced. Word stimuli for these dimensions were derived from Kurdi et al. ^[1]^ (see Table S12 for the full word list). In the demographic group SC-IAT study, participants completed four SC-IATs (counterbalanced) measuring the representation of Gender, Age, Social Class, and Race. Word stimuli for these demographic groups were developed by integrating terms from Charlesworth et al. ^[6]^ and Kurdi et al. ^[1]^ (see Table S12 for the full word list).

Each SC-IAT task followed a standard four-block design divided into two stages ^[8]^, each consisting of one practice block and one test block. In the first stage, participants categorized the target and one attribute (e.g., AI + smart) using the same response key (“e”), while the opposite attribute (e.g., dumb) was assigned to a different key (“i”). In the second stage, key pairings were reversed. To control for order effects, block order was counterbalanced across participants following Greenwald et al. ^[11]^ The number of trials in the practice and test blocks was proportional to the total number of target and evaluative category words, calculated as the sum of target and evaluative words multiplied by two and six, respectively. For example, because the dictionaries for AI, men, and women contained five, four, and four words, respectively, the practice block in the SC-IAT for men-women included (5 + 4 + 4) × 2 = 26 trials. Following Greenwald et al., ^[12]^ incorrect key responses triggered a red “×” as error feedback, displayed for 300 ms. For correct responses, a blank screen was shown for 300 ms before presenting the next stimulus. While there was no maximum response time limit for each trial, participants with unusually fast or slow responses were excluded based on the data processing procedure outlined by Krapinski and Steinman. ^[8]^

Participants also completed an attention-check question (e.g., “Please select ‘agree/disagree’”) randomly embedded within the task.

***Explicit Measures***

In both validation studies, participants’ explicit representations of AI were assessed using two 7-point Likert scales adapted from prior research. The first one, adapted from Greenwald et al., ^[13]^ asked participants to rate the extent to which they associated specific attributes with AI. In the warmth-competence SC-IAT study, these attributes included “warm,” “cold,” “competent,” and “incompetent.” In the demographic group SC-IAT study, attributes related to demographic groups included “man,” “woman,” “young,” “old,” “rich,” “poor,” “White,” and “Asian.” Next, we calculated pairwise differences between the attributes to obtain the difference scores between the target (i.e., AI) and paired attributes. For instance, the warmth-related score was computed as the difference between participants’ ratings of “warm” and “cold.”

The second one, adapted from Kurdi et al., ^[1]^ asked participants to answer direct questions to assess their relative explicit beliefs about AI. For example, in the competence–warmth SC-IAT study, participants were asked, “Do you consider most artificial intelligences to be warm or cold?” In demographic group SC-IAT study, a sample item was, “Do you consider most artificial intelligences to be masculine or feminine?”

We calculated the comprehensive explicit score for each SC-IAT by averaging responses from the two explicit measures. For the competence–warmth SC-IAT study, this score reflected participants’ overall stereotypical representations of AI in terms of high versus low warmth and competence. Similarly, for the demographic group SC-IAT study, the explicit score captured participants’ stereotypical representations of AI in relation to advantaged versus disadvantaged demographic groups such as gender, age, socioeconomic status, and race.

***Data Analysis***

Statistical analyses of this study were performed using R (version 4.4.2; R Core Team, 2024, RRID: SCR_001905), with statistical significance set at *p* < .05. Effect sizes, including Cohen’s *d* and 95% CI, were reported where applicable.

SC-IAT data were processed using the standard algorithm and exclusion criteria recommended by Karpinski & Steinman. ^[8]^

**Results**

We provided the demographic information in Table S13.

***Validation Results for Study 1***

In competence–warmth SC-IAT study, the target category was AI, while the attribute categories were high versus low competence and high versus low warmth. Following Greenwald et al., ^[13]^ a difference score (D-score) for each SC-IAT were calculated, with positive scores (D > 0) indicating stronger associations with high competence/warmth and negative scores (D < 0) indicating the opposite. The SC-IAT results revealed that participants implicitly associated AI more strongly with high competence than with low competence (*t*(98) = 9.903, *p* < 0.001, Cohen’s *d* = 0.995, 95%CI [0.796, 1.195]) and more strongly with high warmth than with low warmth (*t*(98) = 5.110, *p* < 0.001, Cohen’s *d* = 0.514, 95%CI [0.314, 0.713]). The same was true for explicit measures, which indicated that participants explicitly associated AI more strongly with high competence than with low competence (*t*(98) = 68.927, *p* < 0.001, Cohen’s *d* = 6.927, 95%CI [6.728, 7.127]) and more strongly with high warmth than with low warmth (*t*(98) = 41.220, *p* < 0.001, Cohen’s *d* = 4.143, 95%CI [3.943, 4.342]).

***Validation Results for Study 2***

The demographic group SC-IAT study extended the investigation of Study 2 to implicit and explicit associations using SC-IAT and self-report measures. SC-IAT results revealed stronger implicit associations of AI with Men (*t*(96) = 5.268, *p* < 0.001, Cohen’s *d* = 0.535, 95%CI [0.333, 0.736]), Young (*t*(96) = 5.711, *p* < 0.001, Cohen’s *d* = 0.580, 95%CI [0.378, 0.781]), Rich (*t*(96) = 7.581, *p* < 0.001, Cohen’s *d* = 0.770, 95%CI [0.568, 0.971]), and White (*t*(96) = 3.064, *p* = 0.003, Cohen’s *d* = 0.311, 95%CI [0.110, 0.513]), compared to their disadvantaged counterparts. Explicit measures mirrored these patterns (Men: *t*(96) = 14.820, *p* < 0.001, Cohen’s *d* = 1.505, 95%CI [1.303, 1.706]; Young: *t*(96) = 47.920, *p* < 0.001, Cohen’s *d* = 4.866, 95%CI [4.664, 5.067]; Rich: *t*(96) = 32.736, *p* < 0.001, Cohen’s *d* = 3.324, 95%CI [3.122, 3.525]; White: *t*(96) = 11.048, *p* < 0.001, Cohen’s *d* = 1.122, 95%CI [0.920, 1.323]).

We also conducted internal meta-analyses across validation studies, reaving a significant big effect (*d* = 2.070, *p* < 0.001, 95% CI [0.895, 3.245]; see Figure S7)

# Study 3

**Method Details of FMAT**

In part of Study 3, we calculated the stereotypical representation of different demographic groups in the dimensions of competence and warmth using the FMAT, identical to those in Studies 1b and 2b. The query templates used in Study 3 are displayed in Table S5. The [MASK] token was replaced with words related to demographic groups. We selected representative words from each group in the dictionary of Studies 1b and 2b as target [MASK] words (Table S14). Additionally, the {ATTRIB} token was replaced by high and low pairs within each dimension of warmth and competence, identical to those used in Study 1b.

We also analyzed the reliability of FMAT calculation. The inter-rater agreement among the 30 BERT language models ICC_average_ = 0.934; for all query templates in singular form (i.e. three queries), the ICCs were high, ranging from 0.921 to 0.935, and for all query templates in plural form (i.e. four queries), the ICCs were also high, ranging from 0.921 to 0.932 (Figure S8).

Second, we calculated the internal consistency of LPRs across query templates in both singular (*k* = 3) and plural (*k* = 4) forms, separately for each demographic group. The results indicated good internal consistency among the queries (α_query (singular forms)_ = 0.870–0.906; α_query (plural forms)_ = 0.878–0.902).

# Study 4

**Method Details**

***Occupation Lists***

We used the same occupation list as in Hofmann et al. ^[14]^ Following their analysis code, we obtained 65 occupations along with their associated prestige scores. These scores were derived from a dataset based on the 2012 US General Social Survey, ^[15]^ which measures occupational prestige on a scale from 1 (low prestige) to 9 (high prestige). The final set of occupations included in the analysis are as follows: *accountant, actor, actress, administrator, analyst, architect, artist, assistant, astronaut, athlete, attendant, author, broker, chef, chief, cleaner, clergy, clerk, collector, cook, counselor, curator, dentist, designer, detective, developer, director, doctor, driver, economist, editor, engineer, farmer, guard, inspector, instructor, journalist, judge, landlord, lawyer, manager, mechanic, minister, model, musician, nurse, operator, photographer, physician, pilot, priest, professor, psychiatrist, psychologist, scientist, secretary, soldier, student, supervisor, surgeon, tailor, teacher, technician, veterinarian, writer.*

***A Complete Experiment Process***

For each experiment, we applied a random sampling process to select 20 occupations from the full list of 65 occupations for each analysis iteration. The function of *logit_bias* is banned mainly because it allows attackers to query the API and extract the model’s embedding layers, which could expose the internal structure of the model and raise security concerns. ^[16]^ Due to limitations with the *logit_bias* parameter by OpenAI for controlling output, we assigned each occupation a unique numerical ID (number 1–20) to avoid potential biases arising from word prefixes, suffixes, or other structural features that could influence output quality. This numeric assignment ensured consistency and helped eliminate any biases linked to the structure of the words. The occupation-ID mappings were stored in a JSON format (e.g., {“teacher”: 1, “clerk”: 2}) to ensure consistency and traceability across analyses. Each occupation was then embedded into both the benefit and replacement prompts.

***Prompts***

We designed two sets of prompts to capture the dual impacts of AI on different occupations, drawing on multiple AI-related scales for reference. ^[17-19]^ One set focused on identifying occupations that benefit from AI, while the other aimed to identify occupations that may be replaced by AI (see Table S15). This approach allows us to compare between occupations that are viewed as being enhanced by AI technology versus those that are seen as vulnerable to automation, providing a more comprehensive understanding of AI’s potential effects on the workforce.

An example prompt is as follows:

*Each word in the following word list has a unique number. Please select one word from this word list to fill in the {MASK} in the text below.*

*{ “teacher**”: 1, “clerk”: 2, “instructor”: 3, “actress”: 4, “driver”: 5, “supervisor”: 6, “musician”: 7, “accountant”: 8, “developer”: 9, “mechanic”: 10, “scientist”: 11, “journalist”: 12, “student”: 13, “lawyer”: 14, “photographer”: 15, “cleaner”: 16, “attendant”: 17, “collector”: 18, “clergy”: 19, “priest”: 20}*

*The occupation that is more likely to benefit from Artificial Intelligence is {MASK}.*

***Sampling***

To ensure robust sampling, the experiment process was repeated, ensuring that each occupation appeared at least 100 times across both the benefit and replacement prompts. This repetition allowed for a robust analysis of each occupation’s association with AI in both contexts (benefit and replacement). We used the *response_format* parameter^^[[1]](#footnote-1)^^ to restrict the output format to a JSON string ({“score”: int}) and obtained the probability $P(x|v(t))$ of each occupation being filled into the {MASK} position using the *logprobs* parameter1. Finally, we mapped the occupation IDs back to the original occupation names and calculated the average probability for each occupation.

# References

1. B. Kurdi, T. C. Mann, T. E. Charlesworth, M. R. Banaji, *Proc. Natl. Acad. Sci. U.S.A.* **2019**, *116*, 5862–5871.

2. G. Nicolas, X. Bai, S. T. Fiske, *Eur. J. Soc. Psychol*. **2021**, *51*, 178–196.

3. H. W. S. Bao, *J. Pers. Soc. Psychol*. **2024**, *127*, 537–561.

4. J. Pinheiro, D. Bates, R Core Team, *CRAN: nlme citation info*. **2025**.

5. T. A. B. Snijders, R. J. Bosker, *Multilevel Analysis: An Introduction to Basic and Advanced Multilevel Modeling*, SAGE, New Delhi **2011**.

6. T. E. Charlesworth, A. Caliskan, M. R. Banaji, *Proc. Natl. Acad. Sci. U.S.A*. **2022**, *119*, e2121798119.

7. G. A. Chen, J. Y. Buell, *Race Ethn. Educ.* **2018**, *21*, 607–625.

8. A. Karpinski, R. B. Steinman, *J. Pers. Soc. Psychol*. **2006**, *91*, 16–32.

9. F. Faul, E. Erdfelder, A. G. Lang, A. Buchner, *Behav. Res. Methods.* **2007**, *39*, 175–191.

10. B. A. Nosek, A. G. Greenwald, M. R. Banaji, *Pers. Soc. Psychol. Bull.* **2005**, *31*, 166–180.

11. A. G. Greenwald, M. Brendl, H. Cai, D. Cvencek, J. F. Dovidio, M. Friese, A. Hahn, E. Hehman, W. Hofmann, S. Hughes, I. Hussey, C. Jordan, T. A. Kirby, C. K. Lai, J. W. B. Lang, K. P. Lindgren, D. Maison, B. D. Ostafin, J. R. Rae, K. A. Ratliff, A. Spruyt, R. W. Wiers, *Behav. Res. Methods.* **2021**, *54*, 1161–1180.

12. A. G. Greenwald, D. E. McGhee, J. L. Schwartz, *J. Pers. Soc. Psychol*., **1998**, *74*, 1464–1480.

13. A. G. Greenwald, B. A. Nosek, M. R. Banaji, *J. Pers. Soc. Psychol*., **2003**, *85*, 197–216.

14. V. Hofmann, P. R. Kalluri, D. Jurafsky, S. King, *Nature*, **2024**, *633*, 147–154.

15. T. W. Smith, J. Son, *Measuring occupational prestige on the 2012 general social survey*, NORC at the University of Chicago, Chicago, IL. **2014**.

16. N. Carlini, D. Paleka, K. D. Dvijotham, T. Steinke, J. Hayase, A. F. Cooper, K. Lee, M. Jagielski, M. Nasr, A. Conmy, I. Yona, E. Wallace, D. Rolnick, F. Tramèr, *Stealing part of a production language model.* arXiv: 2403.06634v2. **2024**.

17. S. Hovick, V. S. Freimuth, A. Johnson‐Turbes, D. D. Chervin, *Risk Anal*. **2011**, *31*, 1789-1799.

18. W. Wang, J. Downey, F. Yang, *Glob. Media China.* **2023**, *0*, 1–17.

19. Y. Y. Wang, Y. S. Wang, *Interact. Learn. Environ*. **2019**, *30*, 619–634.

Table S1. AI-Related Words Used in Studies 1, 2, and 3.

| **Target** | **Forms** | **Case sensitive** | **Words** |
| --- | --- | --- | --- |
| Artificial Intelligence | Singular forms | Cased | Artificial Intelligence, AI Agent, Intelligent Agent, Robot |
|  |  | Uncased | artificial intelligence, ai agent, intelligent agent, robot |
|  | Plural forms | Cased | Artificial Intelligences, AI Agents, Intelligent Agents, Robots |
|  |  | Uncased | artificial intelligences, ai agents, intelligent agents, robots |

Table S2. Dictionary of Competence and Warmth for the SC-WEAT in Study 1a.

| **Dictionary** | **Attribute category** | **Words** |
| --- | --- | --- |
| Kurdi et al. | High competence | able, capable, competent, confident, efficient, intelligent, proficient, qualified, skilled, skillful, smart |
|  | Low competence | dumb, foolish, helpless, ignorant, incompetent, inefficient, inept, unqualified, uncertain, unintelligent, unskilled |
|  | High warmth | agreeable, dependable, friendly, good-natured, kind, nice, sincere, supportive, trustworthy, warm, well-intentioned |
|  | Low warmth | cold, deceitful, disloyal, dishonest, hateful, hostile, mean, selfish, unfriendly, untrustworthy, vicious |
| Nicolas et al. | High competence | confident, assertive, independent, active, determined, wealthy, powerful, superior, influential, successful,… |
|  | Low competence | different, unassertive, dependent, inactive, doubtful, poor, powerless, inferior, uninfluential, unsuccessful,… |
|  | High warmth | sociable, friendly, warm, liked,outgoing, moral, trustworthy, sincere, fair, tolerant, competent, competitive, intelligent, able, educated,… |
|  | Low warmth | unsociable, unfriendly, cold, disliked, shy, immoral, untrustworthy, insincere, unfair, intolerant, incompetent, competitive, unintelligent, unable, uneducated,… |

Table S3. Summary of the Word Embedding Models Sampled in Studies 1a, 2a, 3.

| **Algorithm** |  | **Corpora** | | | | | | |
| --- | --- | --- | --- | --- | --- | --- | --- | --- |
|  |  | **Training dataset** |  | **Case-sensitive** |  | **Token size** |  | **Vocabulary size** |
| Glove |  | Common Crawl |  | Yes |  | 840 billion |  | 2.2 million |
|  |  | Common Crawl |  | No |  | 42 billion |  | 1.9 million |
|  |  | Wikipedia 2014, Giga5 |  | No |  | 27 billion |  | 1.2 million |
| FastText |  | Wikipedia 2017, UMBC webbase corpus, and statmt.org news dataset |  | No |  | 16 billion |  | 1 million |
|  |  | Common Crawl |  | No |  | 600 billion |  | 2 million |

*Note*. All embeddings have 300 dimensions

Table S4. Summary of the BERT Language Models Sampled in Studies 1a, 2a, 3.

| **Model name** | **Model architecture** | **Model size** | **Corpora** |
| --- | --- | --- | --- |
| bert-base-uncased | BERT | 109.5 million | WIKI, BOOK |
| bert-base-cased | BERT | 108.3 million | WIKI, BOOK |
| bert-large-uncased | BERT | 335.1 million | WIKI, BOOK |
| bert-large-cased | BERT | 333.6 million | WIKI, BOOK |
| bert-large-uncased-whole-word-masking | BERT | 335.1 million | WIKI, BOOK |
| bert-large-cased-whole-word-masking | BERT | 333.6 million | WIKI, BOOK |
| albert-base-v1 | ALBERT | 11.7 million | WIKI, BOOK |
| albert-base-v2 | ALBERT | 11.7 million | WIKI, BOOK |
| albert-large-v1 | ALBERT | 17.7 million | WIKI, BOOK |
| albert-large-v2 | ALBERT | 17.7 million | WIKI, BOOK |
| albert-xlarge-v1 | ALBERT | 58.7 million | WIKI, BOOK |
| albert-xlarge-v2 | ALBERT | 58.7 million | WIKI, BOOK |
| distilbert-base-uncased | DistilBERT | 66.4 million | WIKI, BOOK |
| distilbert-base-cased | DistilBERT | 65.2 million | WIKI, BOOK |
| roberta-base | RoBERTa | 124.6 million | BOOK, CC-NEWS, OPEN, STORIES |
| distilroberta-base | RoBERTa | 82.1 million | OPEN |
| muppet-roberta-base | RoBERTa | 124.6 million | WIKI, BOOK |
| bart-base | BART | 139.4 million | BOOK, CC-NEWS, OPEN, STORIES |
| bart-large | BART | 406.3 million | BOOK, CC-NEWS, OPEN, STORIES |
| electra-base-generator | ELECTRA | 33.5 million | WIKI, BOOK |
| electra-large-generator | ELECTRA | 51.0 million | WIKI, BOOK, ClueWeb, CC*, Giga5 |
| mobilebert-uncased | MobileBERT | 24.6 million | WIKI, BOOK |
| base-mlm-tweet | BERT | 109.5 million | IMDB |
| bertweet-base | RoBERTa | 134.9 million | TWITTER |
| bertweet-covid19-base-uncased | RoBERTa | 134.9 million | TWITTER (COVID-19) |
| bertweet-covid19-base-cased | RoBERTa | 134.9 million | TWITTER (COVID-19) |
| twitter-roberta-base | RoBERTa | 124.6 million | TWITTER |
| twitter-roberta-base-2019-90m | RoBERTa | 124.6 million | TWITTER |
| twitter-roberta-base-2021-124m | RoBERTa | 124.6 million | TWITTER |
| twitter-roberta-base-2022-154m | RoBERTa | 124.6 million | TWITTER |

*Note*. (i) Model size refers to the number of parameters in each model during pretraining. (ii) Abbreviations for training corpora: WIKI: English Wikipedia (2.5 billion words); BOOK: BookCorpus (11,038 unpublished books scraped from the internet; 800 million words); CC: CommonCrawl (a vast corpus of publicly available web data); CC-NEWS: English portion of the CommonCrawl News dataset (63 million English news articles); OPEN: OpenWebText (8 million documents from Reddit); TWITTER: 850 million English tweets; STORIES: A subset of CommonCrawl (1 million documents, primarily stories); ClueWeb: ClueWeb 2012 web document collection (733 million web pages); Giga5: English Gigaword Fifth Edition (a comprehensive archive of newswire text data; 4 million words); TWITTER (COVID): 850 million English tweets, containing 845 million Tweets streamed from 01/2012 to 08/2019 and 5 million Tweets related to the COVID-19 pandemic; IMDB: A large movie review dataset (50,000 movie reviews). (iii) The * mark indicates the use of a portion of the Common Crawl dataset, containing approximately 60 billion words. (iv) The term “BERT models” or simply "models" (rather than "BERT-based language models") is used when the distinction is contextually irrelevant. (v) For simplicity, all 30 models are collectively referred to as “BERT language models” or “BERT-based models” even though some (e.g., BART, ELECTRA) differ from the original BERT framework.

Table S5. Query Templates Used in Studies 1b and 3.

| **Forms** | ***N*** | **Query templates** |
| --- | --- | --- |
| Singular forms | 4 | “[MASK] is {ATTRIB}.”  **“The [MASK] is {ATTRIB}.”**  **“This [MASK] is {ATTRIB}.”**  **“That [MASK] is {ATTRIB}.”** |
| Plural forms | 5 | “[MASK] are {ATTRIB}.”  **“The [MASK] are {ATTRIB}.”**  **“These [MASK] are {ATTRIB}.”**  **“Those [MASK] are {ATTRIB}.”**  **“Most [MASK] are {ATTRIB}.”** |

*Note.* Bolded query templates are used in Study 3 to generate four-dimensional representations for each demographic group based on warmth and competence. [MASK] = Al related words; {ATTRlB} = high and low pairs within each dimension of warmth and competence.

Table S6. Dictionary of Competence and Warmth for the FMAT in Study 1b.

| **Dictionary** | **Dimension of attributes** | **Number of pairs** | **Pairwise contrast {ATTRIB}** | |
| --- | --- | --- | --- | --- |
|  |  |  | High | Low |
| Bao & Gries (2024) | Competence | 24 pairs | able  capable  clever  competent  competitive  intelligent  knowledgeable  skilled  skillful  smart  sophisticated  wise  active  adventurous  ambitious  assertive  brave  certain  confident  decisive  determined  dominant  independent  proactive | unable  incapable  foolish  incompetent  uncompetitive  unintelligent  unknowledgeable  unskilled  unskillful  stupid  unsophisticated  unwise  inactive  unadventurous  unambitious  unassertive  afraid  uncertain  unconfident  indecisive  undetermined  submissive  dependent  passive |
|  | Warmth | 24 pairs | affectionate  agreeable  amiable  amicable  friendly  likable  outgoing  responsive  sociable  social  supportive  warm  authentic  ethical  faithful  honest  loyal  moral  reliable  responsible  sincere  tolerant  trustworthy  truthful | unaffectionate  disagreeable  unamiable  unamicable  unfriendly  unlikable  shy  unresponsive  unsociable  unsocial  unsupportive  cold  inauthentic  unethical  unfaithful  dishonest  disloyal  immoral  unreliable  irresponsible  insincere  intolerant  untrustworthy  untruthful |
| Kurdi et al. (2019) | Competence | 11 pairs | friendly  well-intentioned  trustworthy  warm  good-natured  sincere  nice  kind  dependable  agreeable  supportive | unfriendly  hateful  untrustworthy  cold  mean  dishonest  vicious  deceitful  disloyal  selfish  hostile |
|  | Warmth | 11 pairs | competent  confident  capable  efficient  intelligent  skillful  skilled  qualified  proficient  able  smart | incompetent  uncertain  ignorant  inefficient  unintelligent  helpless  unskilled  unqualified  inept  dumb  foolish |

Table S7. Internal Consistency (Cronbach’s *α* for Queries) and Effect Sizes (*d*) of the FMAT in Both Singular and Plural Forms in Study 1b (Results from Linear Regression).

| **Dictionary** | **Contrast attributes** | **α_query_** | |  | **Effect size** | | |
| --- | --- | --- | --- | --- | --- | --- | --- |
|  |  | **α_query (singular forms)_**  **(*k* = 4)** | **α_query (plural forms)_**  **(*k* = 5)** |  | **Overall** | **Singular form** | **Plural form** |
| Bao & Gries (2024) | Competence (high vs. low) | 0.880 | 0.886 |  | 0.097***  [0.088, 0.106] | 0.104***  [0.091, 0.117] | 0.089***  [0.077, 0.101] |
|  | Warmth  (high vs. low) | 0.874 | 0.883 |  | –0.098***  [–0.107, –0.089] | –0.060***  [–0.073, –0.047] | –0.136***  [–0.148, –0.124] |
| Kurdi et al. (2019) | Competence (high vs. low) | 0.865 | 0.906 |  | 0.311***  [0.298, 0.324] | 0.340***  [0.321, 0.360] | 0.281***  [0.264, 0.299] |
|  | Warmth  (high vs. low) | 0.852 | 0.891 |  | –0.279***  [–0.292, –0.265] | –0.245***  [–0.265, –0.226] | –0.312***  [–0.329, –0.294] |

Table S8. Dictionary of Demographic Groups for the SC-WEAT in Study 2a.

| **Social category** | **Attribute category** | **Words** |
| --- | --- | --- |
| Gender | Man | men, man, male, males, masculine, masculinity, he, him, his, himself, mr., mister, boy, boys, guy, guys, fella, fellas, gent, gents, sir, sirs, bloke, blokes, gentleman, gentlemen, lad, lads, king, kings, prince, princes, manly |
|  | Woman | women, woman, female, females, feminine, femininity, she, her, hers, herself, girl, gal, gals, mrs., ms., miss., missus, mz., lady, ladies, dame, dames, bride, brides, gentlewoman, gentlewomen, lass, lassie, maiden, maidens, madam, queen, queens, princess, princesses, womanly |
| Age | Young | young, youth, youthful, junior, juniors, child, adolescent, children, youngster, adolescents, descendent, descendants |
|  | Old | elderly, elder, elders, senior, seniors, grandparent, grandmother, grandfather, grandparents, grandmothers, grandfathers, ancestor, ancestors, ancestral |
| Social Class | Rich | rich, wealthy, aristocracy, nobility, affluent, moneyed, wealth, aristocrat, aristocrats, prosperous, privileged, bourgeoisie, bourgeois, noble, nobles, nobleman, noblemen, elite, elites, benefactor, benefactors, philanthropist, philanthropists, landowner, landowners |
|  | Poor | poor, needy, destitute, beggars, penniless, miserly, underprivileged, unprivileged, beggar, homeless, impoverished |
| Race | White | whites, caucasian, european, europeans, american, british, english, americans, caucasian, caucasians, englishman, englishmen |
|  | Black | blacks, african, africans, negro, negros, negroid, ethiopian, ethiopians, n*er, n*ers, n*a, n*as |
|  | Asian | oriental, chinese, asiatic, japanese, asian, asians, orientals, chinaman, chinamen, mongolian, mongols |
|  | Irish | irish, celtic, gaelic, celts, irishman, irishmen, dubliner, gael |
|  | Hispanic | hispanos, latinas, mexican, mexicans, hispanic, hispanics, latino, latinos, latina, cuban, cubans, spanish |

Table S9. Query Templates Used in Study 2b.

| **Forms** | ***N*** | **Query templates** |
| --- | --- | --- |
| Singular forms | 4 | “[MASK] {ATTRIB}.”  “The [MASK] {ATTRIB}.”  “This [MASK] {ATTRIB}.”  “That [MASK] {ATTRIB}.” |
| Plural forms | 5 | “[MASK] {ATTRIB}.”  “The [MASK] {ATTRIB}.”  “These [MASK] {ATTRIB}.”  “Those [MASK] {ATTRIB}.”  “Most [MASK] {ATTRIB}.” |

*Note.* Unlike Study 1b, in Study 2b we did not include the verb “be” in the queries. In Study 1b, {ATTRIB} was always an adjective, but in Study 2b, {ATTRIB} includes a wider variety of phrased. Therefore, we incorporated the verb “be” directly into {ATTRIB} for better grammatical integration. [MASK]= Al related words; {ATTRlB} = advantaged demographic and disadvantaged pairwise phrases within each demographic group.

Table S10. Dictionary of Demographic Groups for the FMAT in Study 2b.

| **Forms** | **Group** | **Number of pairs** | **Pairwise contrast {ATTRIB}** | |
| --- | --- | --- | --- | --- |
|  |  |  | Advantaged group | Disadvantaged group |
| Singular forms | Gender | 10 pairs | is a man  is like a man  is manlike  is masculine  is typical of a man  is typical of men  has a masculine personality  has a masculine trait  has masculine characteristics  has masculine traits | is a woman  is like a woman  is womanlike  is feminine  is typical of a woman  is typical of women  has a feminine personality  has a feminine trait  has feminine characteristics  has feminine traits |
|  | Age | 10 pairs | is young  is a youth  is like a youth  is like a young person  is youthful  is typical of the young  has a youthful personality  has a youthful trait  has youthful characteristics  has youthful traits | is old  is an elder  is like an elder  is like an elderly person  is elderly  is typical of the old  has an aged personality  has an elderly trait  has elderly characteristics  has elderly traits |
|  | Social class | 10 pairs | is rich  is the rich  is like the rich  is privileged  is wealthy  is moneyed  is typical of a rich person  is typical of the rich  has a high standard of living  lives a wealthy life | is poor  is the poor  is like the poor  is underprivileged  is impoverished  is penniless  is typical of a poor person  is typical of the poor  has a low standard of living  lives a poor life |
|  | Race | 4 pairs | is like a White person  is like a White person  is like a White person  is like a White person | is like a Black person  ﻿is like a Hispanic person  is like an Asian person  ﻿is like an Irish person |
| Plural forms | Gender | 10 pairs | are men  are like men  are manlike  are masculine  are typical of a man  are typical of men  have a masculine personality  have a masculine trait  have masculine characteristics  have masculine traits | are women  are like women  are womanlike  are feminine  are typical of a woman  are typical of women  have a feminine personality  have a feminine trait  have feminine characteristics  have feminine traits |
|  | Age | 10 pairs | are young  are youths  are like youths  are like young people  are youthful  are typical of the young  have a youthful personality  have a youthful trait  have youthful characteristics  have youthful traits | are old  are elders  are like elders  are like elderly people  are elderly  are typical of the old  have an aged personality  have an elderly trait  have elderly characteristics  have elderly traits |
|  | Social class | 10 pairs | are rich  are the rich  are like the rich  are privileged  are wealthy  are moneyed  are typical of rich people  are typical of the rich  have high standards of living  live a wealthy life | are poor  are the poor  are like the poor  are underprivileged  are impoverished  are penniless  are typical of poor people  are typical of the poor  have low standards of living  live are a poor life |
|  | Race | 4 pairs | are like White people  are like White people  are like White people  are like White people | are like Black people  are like Hispanic people  are like Asian people  are like Irish people |

Table S11. Internal Consistency (Cronbach’s α for queries) and Effect Sizes (d) of the FMAT in Both Singular and Plural Forms in Study 2b (Results from Linear Regression).

| **Contrast demographic groups** | **α_query_** | |  | **Effect size** | | |
| --- | --- | --- | --- | --- | --- | --- |
|  | **α_query (singular forms)_**  **(*k* = 4)** | **α_query (plural forms)_**  **(*k* = 5)** |  | **Overall** | **Singular form** | **Plural form** |
| Gender  (Man vs. Woman) | 0.868 | 0.869 |  | 0.293***  [0.279, 0.306] | 0.285***  [0.265, 0.305] | 0.300***  [0.282, 0.318] |
| Age  (Young vs. Old) | 0.861 | 0.861 |  | 0.136***  [0.122, 0.150] | 0.105***  [0.084, 0.125] | 0.168***  [0.150, 0.186] |
| Social class  (Rich vs. Poor) | 0.834 | 0.903 |  | 0.327***  [0.314, 0.340] | 0.245***  [0.225, 0.265] | 0.409***  [0.391, 0.427] |
| Race  (White vs. Black/Asian/Irish/Hispanic) | 0.759 | 0.894 |  | 0.326***  [0.305, 0.347] | 0.211***  [0.179, 0.243] | 0.441***  [0.412, 0.469] |

Table S12. Word Stimulus for Two Validation Studies.

| **Validation for** | **SC-IAT category** | **Attribute category** | | **Target category (Artificial Intelligence)** |
| --- | --- | --- | --- | --- |
| For Study 1:  2 warmth-competence SC-IATs | AI = High vs. low competence | High competence | competent, confident, capable, efficient, intelligent, skillful, skilled, qualified, proficient, able, smart | Artificial Intelligence, robot, Intelligent Agent, Intelligent Robot, AI |
|  |  | Low competence | incompetent, uncertain, ignorant, inefficient, unintelligent, unskilled, helpless, inept, unqualified, dumb, foolish |  |
|  | AI = High vs. low warmth | High warmth | friendly, well-intentioned, trustworthy, warm, good-natured, sincere, nice, kind, dependable, agreeable, supportive |  |
|  |  | Low warmth | unfriendly, hateful, untrustworthy, cold, mean, dishonest, vicious, deceitful, disloyal, selfish, hostile |  |
| For Study 2:  4 demographic group SC-IATs | AI = Man vs. Woman | Man | men, man, male, he |  |
|  |  | Woman | women, woman, female, she |  |
|  | AI = Young vs. Old | Young | young, youth, junior, youthful, younger |  |
|  |  | Old | old, elderly, senior, elderly, older |  |
|  | AI = Rich vs. Poor | Rich | rich, wealthy, affluent, upper-class |  |
|  |  | Poor | poor, needy, destitute, beggars |  |
|  | AI = White vs. Non-white | White | White, Whites, Europe, Westerner |  |
|  |  | Non-white | Asian, Asians, oriental, Chinese |  |

*Note.* Since our participants were Chinese, only stimuli related to Chinese and Asian individuals were used in the Non-white group.

Table S13. Full Demographic Information in Competence–Warmth SC-IAT Study and Demographic Group SC-IAT Study.

|  | **Final *N*** | | **Age**  (*M* ± *SD*) | **Education level**  (*M* ± *SD*) | **Subjective social status**  (*M* ± *SD*) |
| --- | --- | --- | --- | --- | --- |
|  | **Female** | **Male** |  |  |  |
| Warmth-competence SC-IAT study | 54 | 45 | 29.747±6.223 | 5.131±0.617 | 5.515±1.343 |
| Demographic group SC-IAT study | 62 | 35 | 30.454±7.539 | 5.052±0.508 | 5.557±1.224 |

Table S14. Dictionary of Demographic Groups for the FMAT in Study 3.

| **Demographic group** | **Forms** | Target [MASK] words |
| --- | --- | --- |
| Man | Singular form | man, male |
|  | Plural form | men, males |
| Woman | Singular form | woman, female |
|  | Plural form | women, females |
| Young | Singular form | youth, young person |
|  | Plural form | youths, young people |
| Old | Singular form | elder, old person |
|  | Plural form | elders, old people |
| Rich | Singular form | rich person, privileged person |
|  | Plural form | rich people, privileged people |
| Poor | Singular form | poor person, underprivileged person |
|  | Plural form | poor people, underprivileged people |
| White | Singular form | White person |
|  | Plural form | White people |
| Black | Singular form | Black person |
|  | Plural form | Black people |
| Asian | Singular form | Asian person |
|  | Plural form | Asian people |
| Irish | Singular form | Irish person |
|  | Plural form | Irish people |
| Hispanic | Singular form | Hispanic person |
|  | Plural form | Hispanic people |

Table S15. Prompts in Study 4.

| **Sets** | **Prompts** |
| --- | --- |
| Benefit | 1. The occupation that is more likely to benefit from Artificial Intelligence is {MASK}.  2. The occupation that the adoption of Artificial Intelligence benefits the most is {MASK}.  3. The occupation that benefits the most from Artificial Intelligence is {MASK}.  4. Artificial Intelligence is beneficial to {MASK}.  5. {MASK} gains the most from advancements in Artificial Intelligence.  6. {MASK} is more likely to gain from the use of Artificial Intelligence. |
| Replacement | 1. The occupation that is most likely to be replaced by Artificial Intelligence is {MASK}.  2. The occupation that the adoption of Artificial Intelligence threatens is {MASK}.  3. The occupation that Artificial Intelligence threatens is {MASK}.  4. The occupation that Artificial Intelligence is more likely to replace is {MASK}.  5. The occupation most susceptible to being automated by Artificial Intelligence is {MASK}.  6. {MASK} is more likely to be replaced by Artificial Intelligence. |

Figure S1. Spatial Mapping of AI and Competence–Warmth Attributes.


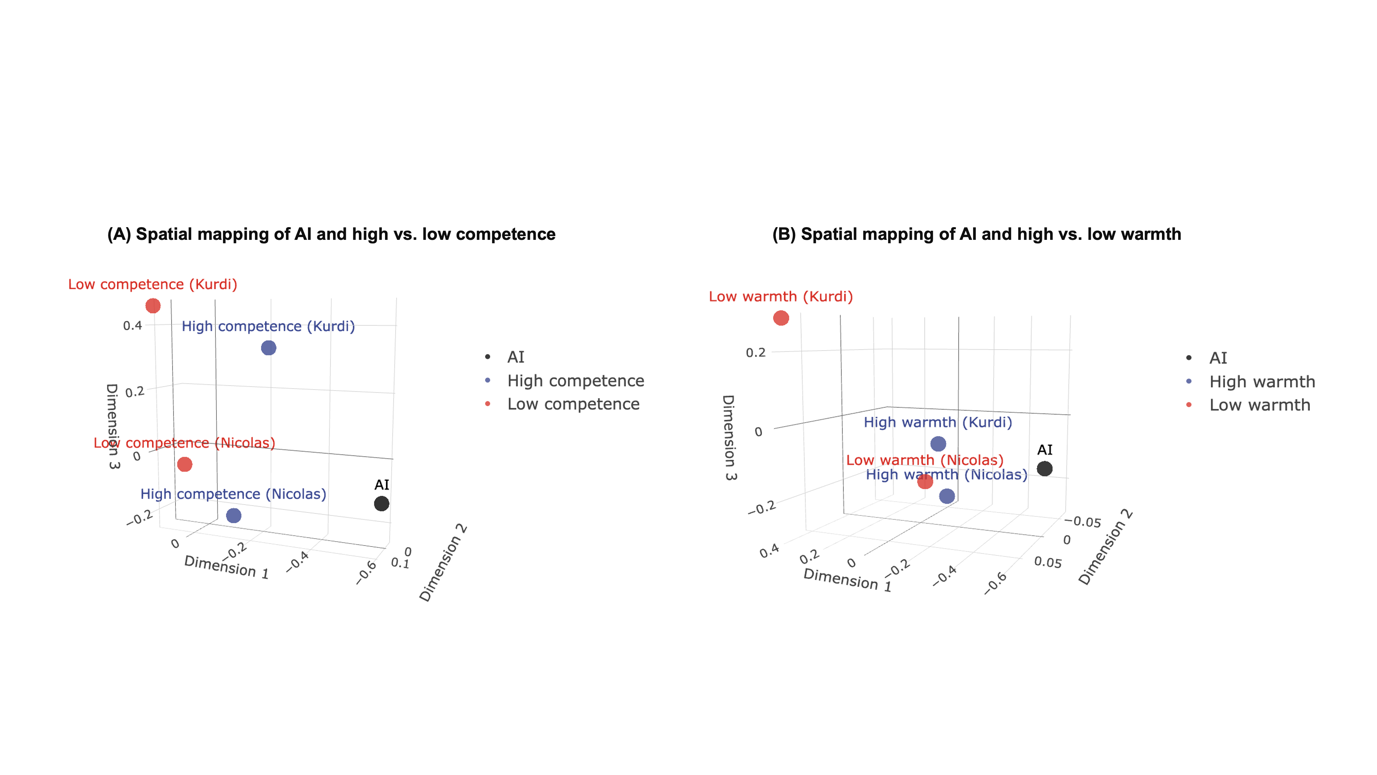


Figure S2. Inter-Rater Agreement (Intraclass Correlation Coefficient, ICC_average_) of the 30 BERT Language Models in Study 1b.

**
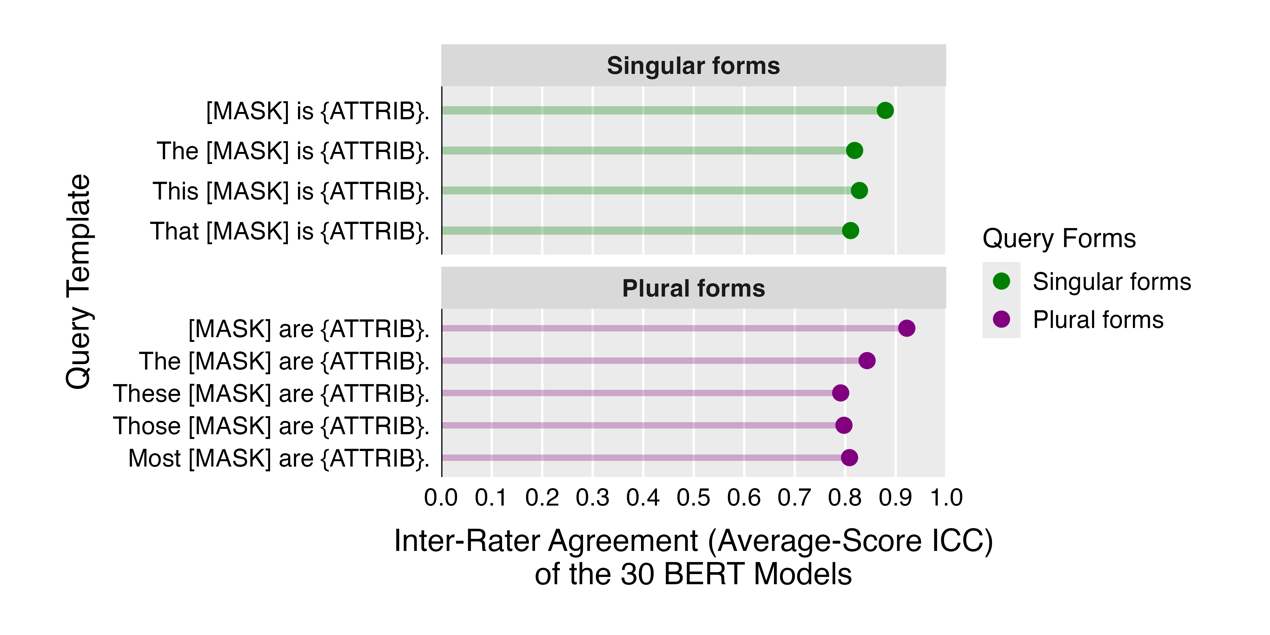
**

Figure S3. Comparison of High vs. Low Competence and Warmth across Query Forms.

**
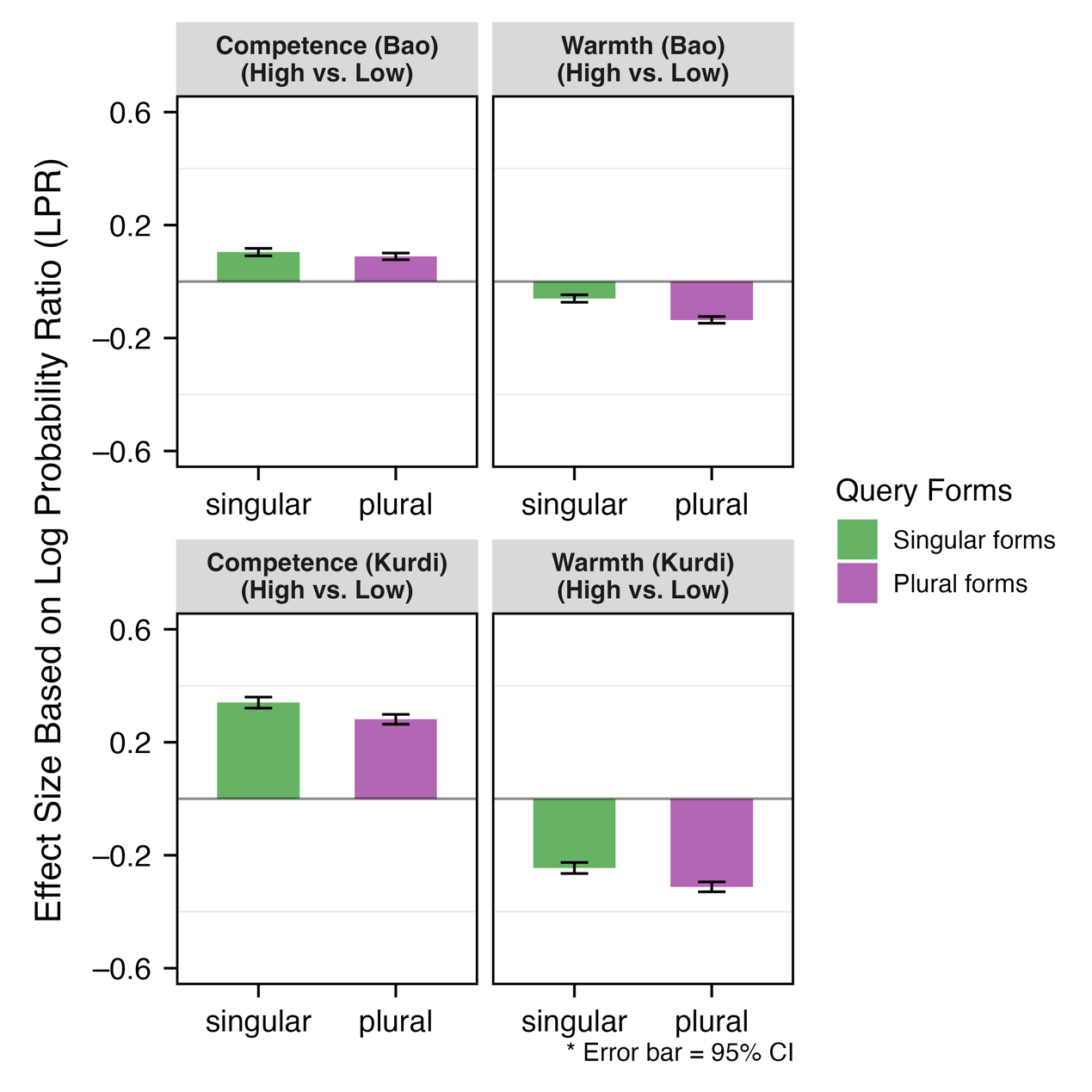
**

Figure S4. Spatial Mapping of AI and Each Demographic Group.

**
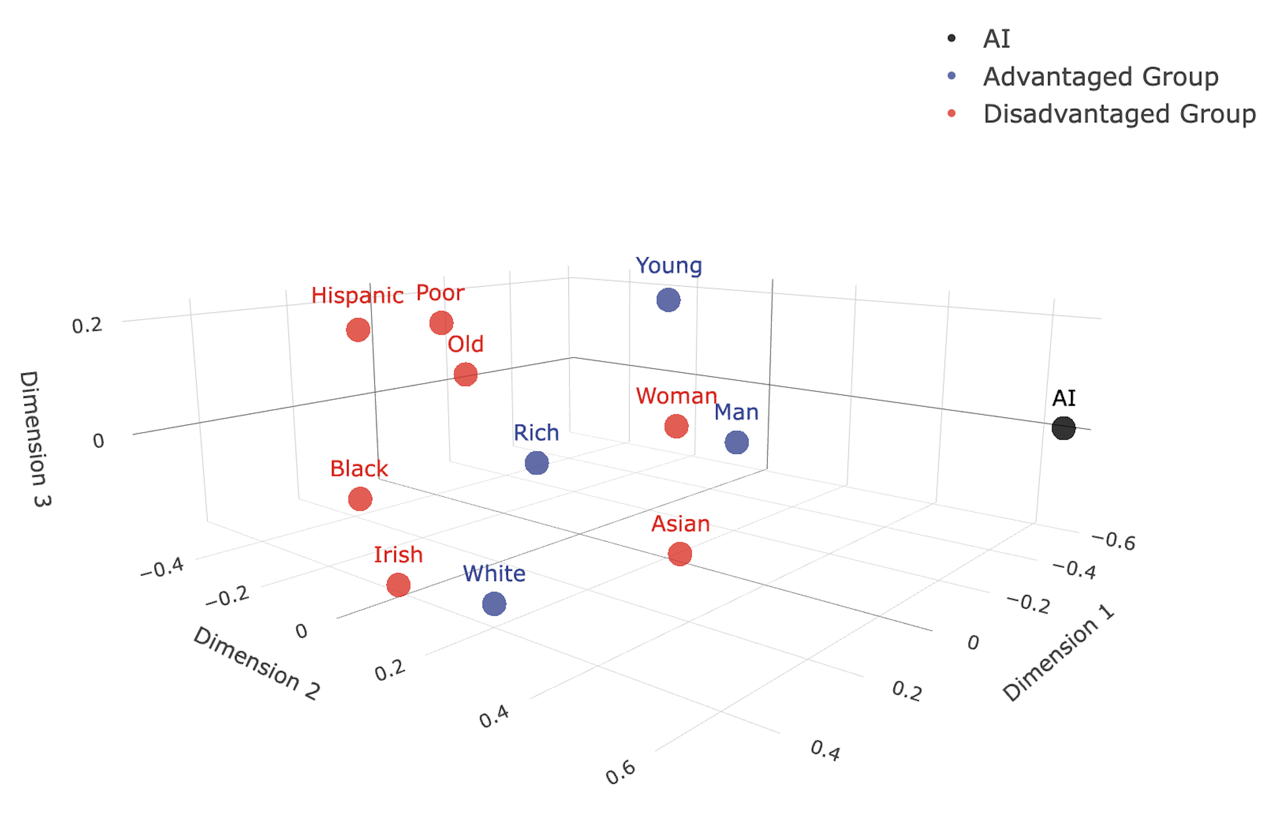
**

Figure S5. Inter-Rater Agreement (Intraclass Correlation Coefficient, ICC_average_) of the 30 BERT Language Models in Study 2b.


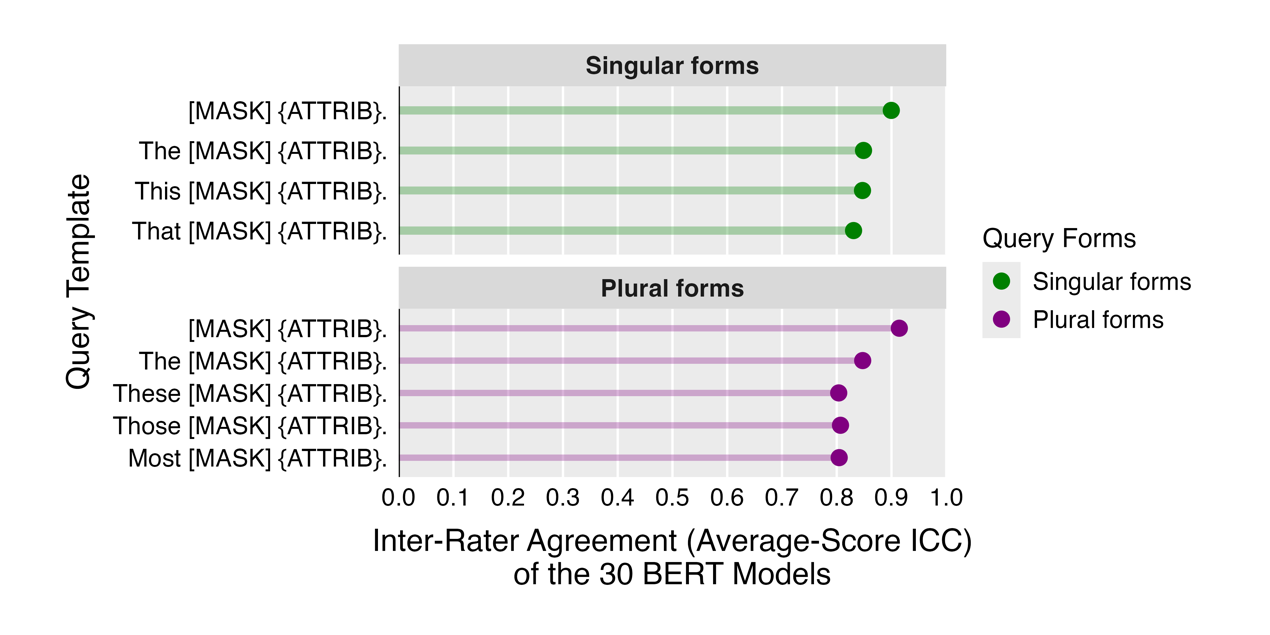


Figure S6. Comparison of Advantaged vs. Disadvantaged Groups across Query Forms.

**
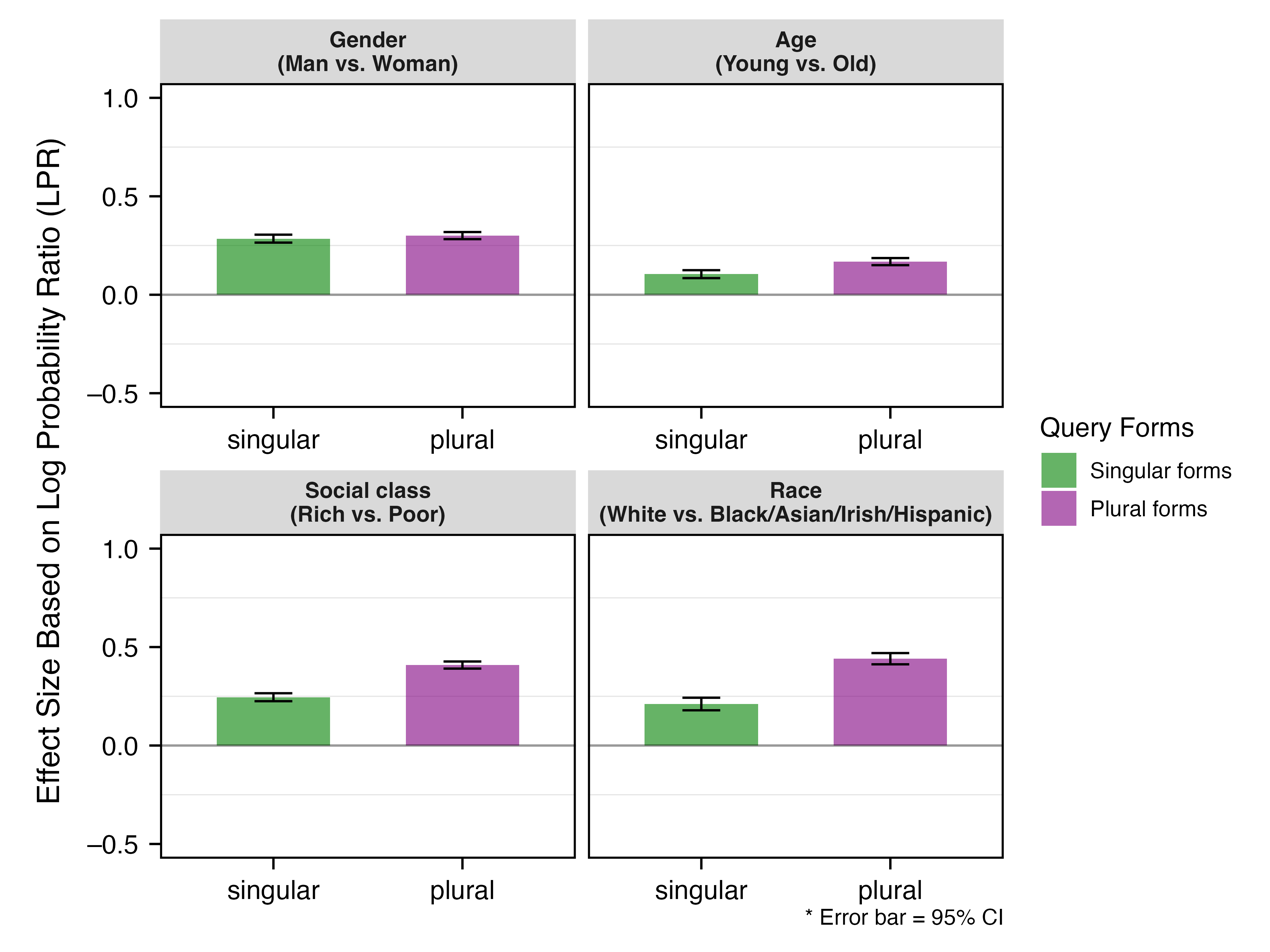
**

Figure S7. Effect sizes across All Validation Studies.


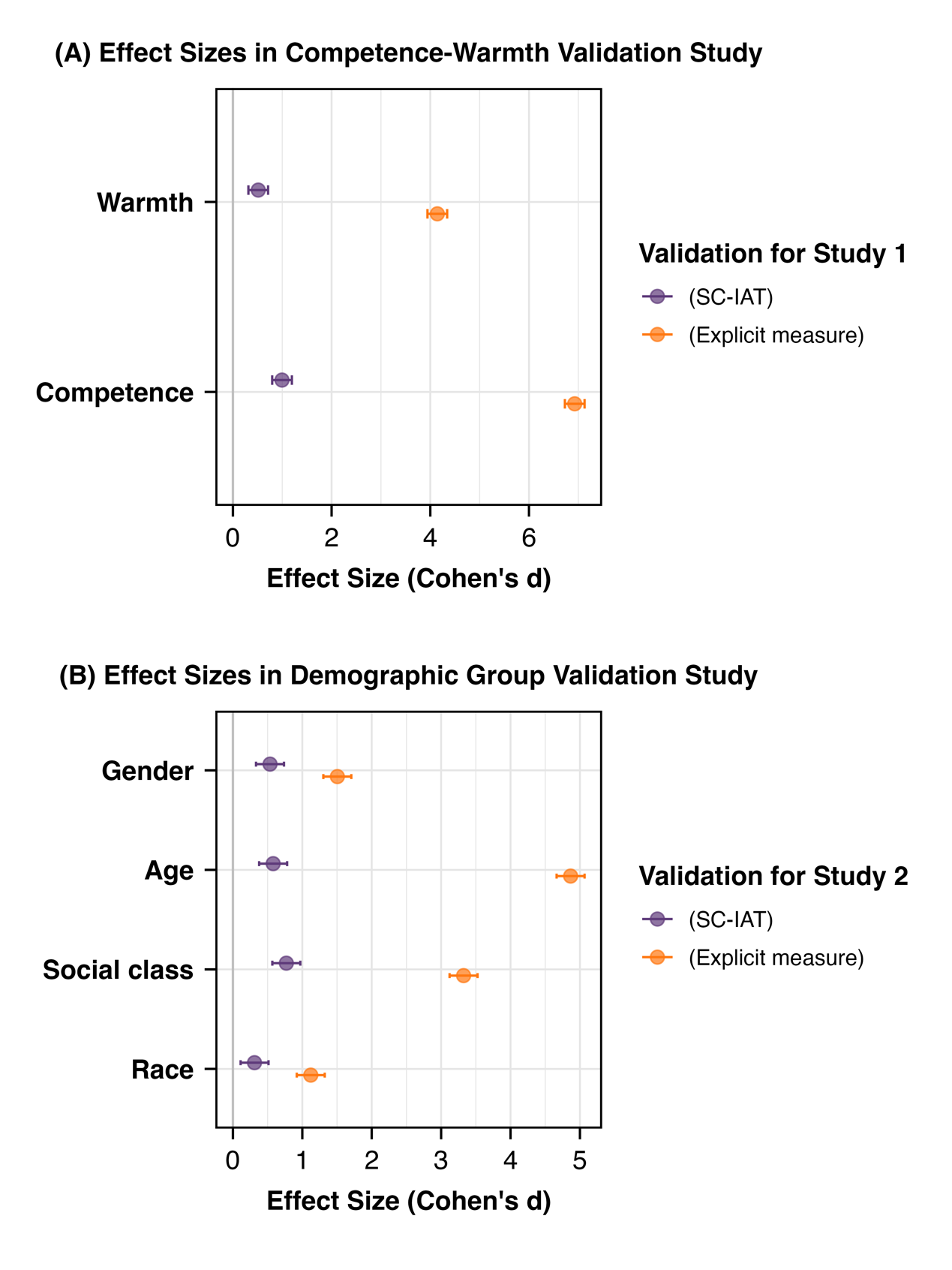


Figure S8. Inter-Rater Agreement (Intraclass Correlation Coefficient, ICC_average_) of the 30 BERT Language Models in Study 3.


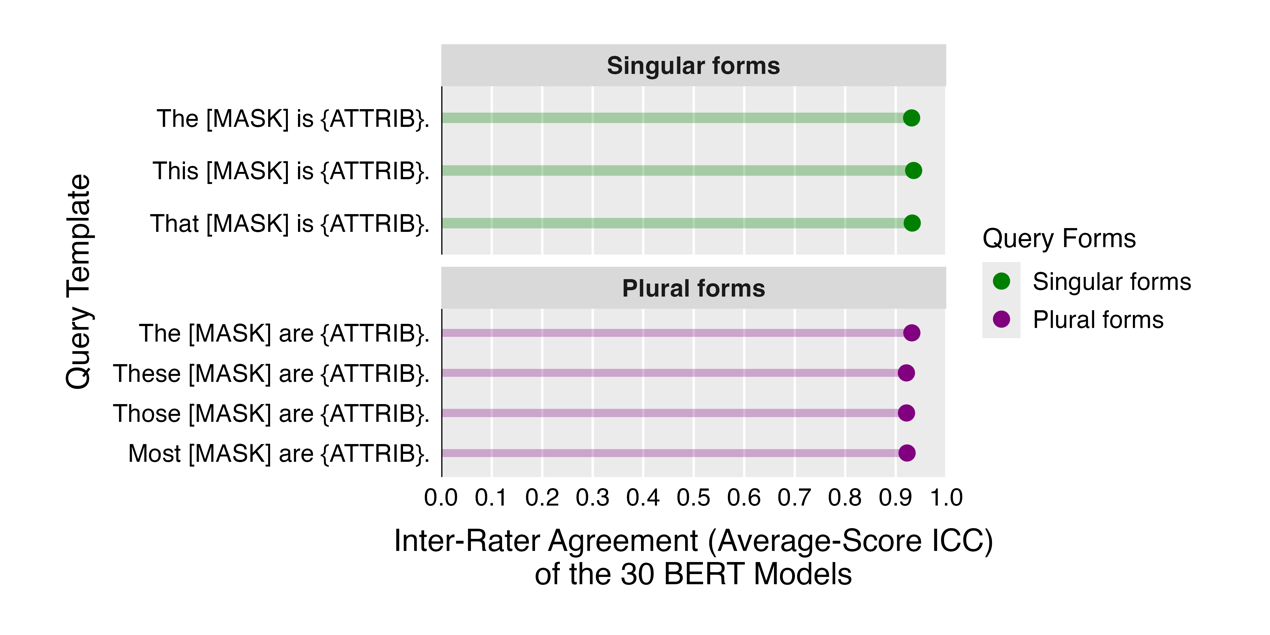


1. These functions are all provided by OpenAI. The *response_format* parameter defines the structure of the model’s output, which can be set to either text or json. When set to json, the response is returned in a structured JSON format, allowing for more detailed parsing and access to specific data elements, such as tokens and metadata. The *logprobs* parameter controls whether log probabilities are included in the response. When set to a positive integer (e.g., *logprobs* = 20), it returns log probabilities for the top *N* tokens at each position in the generated text, providing insight into the model’s confidence in its token predictions. The API is called in Python. For more details, see the OpenAI API Documentation (https://platform.openai.com/docs/api-reference). [↑](#footnote-ref-1)
